# The Expression of *irx7* in the Inner Nuclear Layer of Zebrafish Retina Is Essential for a Proper Retinal Development and Lamination

**DOI:** 10.1371/journal.pone.0036145

**Published:** 2012-04-23

**Authors:** Yuqing Zhang, Yifan Yang, Caleb Trujillo, Wenxuan Zhong, Yuk Fai Leung

**Affiliations:** 1 Department of Biological Sciences, Purdue University, West Lafayette, Indiana, United States of America; 2 Department of Statistics, University of Illinois at Urbana-Champaign, Champaign, Illinois, United States of America; 3 Department of Biochemistry and Molecular Biology, Indiana University School of Medicine Lafayette, Indiana, United States of America; Universidade Federal do Rio de Janeiro, Brazil

## Abstract

*Irx7*, a member in the zebrafish *iroquois* transcription factor (TF) family, has been shown to control brain patterning. During retinal development, *irx7*'s expression was found to appear exclusively in the inner nuclear layer (INL) as soon as the prospective INL cells withdraw from the cell cycle and during retinal lamination. In Irx7-deficient retinas, the formation of a proper retinal lamination was disrupted and the differentiation of INL cell types, including amacrine, horizontal, bipolar and Muller cells, was compromised. Despite *irx7*'s exclusive expression in the INL, photoreceptors differentiation was also compromised in Irx7-deficient retinas. Compared with other retinal cell types, ganglion cells differentiated relatively well in these retinas, except for their dendritic projections into the inner plexiform layer (IPL). In fact, the neuronal projections of amacrine and bipolar cells into the IPL were also diminished. These indicate that the retinal lamination issue in the Irx7-deficient retinas is likely caused by the attenuation of the neurite outgrowth. Since the expression of known TFs that can specify specific retinal cell type was also altered in Irx7-deficient retinas, thus the *irx7* gene network is possibly a novel regulatory circuit for retinal development and lamination.

## Introduction

The vertebrate retina has six types of neuron and one major type of glial cell. All of which originate from the same progenitors in a conserved order [1]. In general, ganglion cells (GCs) are born first, followed by overlapping births of horizontal cells (HCs), cone photoreceptors (cones), amacrine cells (ACs), bipolar cells (BCs), rod photoreceptors (rods) and Muller cells (MCs). During their differentiation, these cell types are organized into three cellular layers, including ganglion cell layer (GCL) that contains primarily GCs, INL that contains ACs, BCs, MCs and HCs, and outer nuclear layer (ONL) that contains rods and cones. The GCL and INL are separated by a synaptic layer called IPL, while INL and ONL are separated by outer plexiform layer (OPL).

The molecular control of retinal differentiation and lamination into three cellular layers [2], is still not clear [3]. Studies in zebrafish have identified key signaling molecules and processes for this control. These include Sonic hedgehog a (Shha) [4], cell adhesion [5], cell polarity regulation [6] and chromatin remodeling [7]. A recent microarray study of chromatin remodeling on zebrafish retinal differentiation have identified 731 genes regulated by Smarca4, a component of chromatin remodeling complex [8]. In Smarca4-deficient retinas, all retinal cell types can be specified and many of them are located in the correct location, but they fail to terminally differentiate [9]; at the same time, retinal lamination is compromised. Thus, Smarca4-regulated genes may play important roles in these terminal differentiation, retinal lamination and patterning processes [8]. For example, several members in the *iroquois* (*irx*) TFs family, including *irx1a*, *3a*, *4a*, *4b*, *5a* and *7*, are transcriptionally activated by Smarca4. These TFs share a similar structure that consists of a homeodomain and an Iro box [10]. Their genomic arrangement is also highly conserved in metazoans, suggesting a conserved function in different species [11]. Indeed, the *irx* genes in both invertebrate and vertebrate have been shown to be an important mediator for embryo patterning. For example, it has been shown that *irx7* regulates the compartmentalization of midbrain and hindbrain [10], [12], [13].

Several *irx* genes are expressed in the retina and regulate its development. These include *irx1a*, *2a*, *3a*, *4a*, *4b*, *5a*, *6a* and *7* in zebrafish [8], [14], [15], [16], [17] and all six *Irx* genes in mouse [18]. Most of these *irx* genes are expressed in GCs region, except for mice *Irx5* [19] that is expressed in GCL and INL, zebrafish *irx6a* that is expressed a subset of outer retinal cells in addition to GCs (http://zfin.org) and zebrafish *irx7* that is only expressed in INL [17]. The regulation of several *irx* genes by Smarca4 in retina [8] hints at the possibility that they may regulate retinal differentiation and lamination. Indeed, the propagation of the Shha neurogenic waves in zebrafish retina [4], [20] is mediated by the expression of *irx1a* and in turn *irx2a* in GCs [15], [16]; and the knockdown of these two *irx* genes compromises retinal differentiation and lamination that resembles the *shha* mutants.

The exclusive expression of zebrafish *irx7* in the INL at 52 hpf [17] is particularly interesting because retinal lamination is established at around this stage. Together with the issues in retinal development in an initial Irx7 knockdown [8], these observations suggest two non-mutually exclusive possibilities. First, *irx7* is essential for retinal patterning and formation of retinal lamination. Second, *irx7* is responsible for INL cells differentiation, which in turn regulates the formation of retinal lamination. The purpose of this study was to define the role of *irx7* in retinal development and lamination. The results indicate that *irx7* is necessary for differentiation of INL and ONL and projection of neuronal processes into the plexiform layers. Compromising these processes may in turn disrupt retinal lamination.

## Results

### *Irx7* is specifically expressed in the INL during retinal development

**Figure 1:**
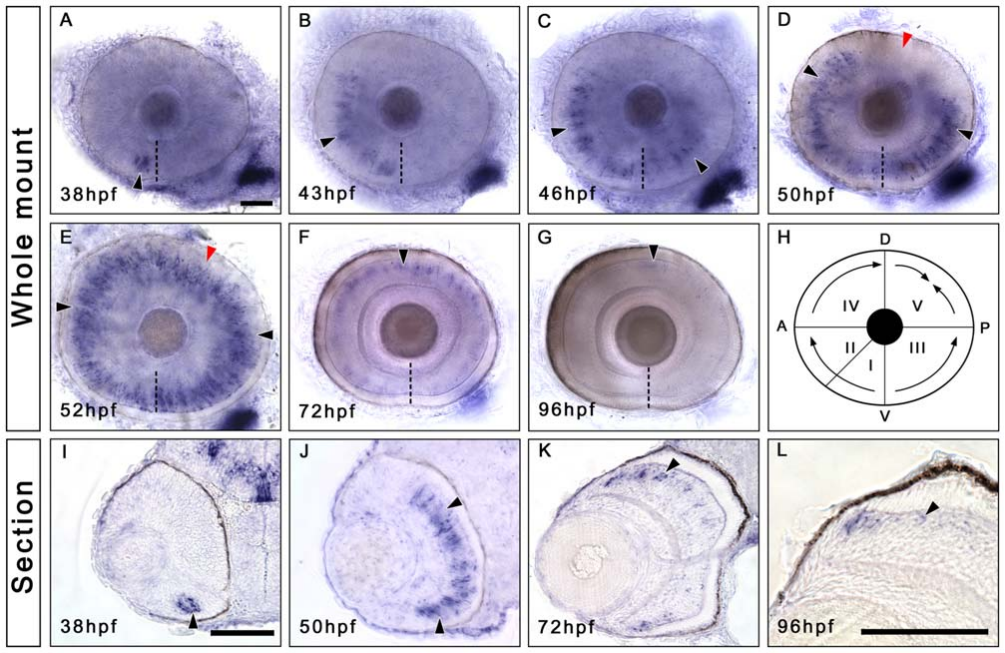
*Irx7* is specifically expressed in the prospective INL during zebrafish retinal development. Whole-mount *in situ* hybridization was conducted to elucidate the expression dynamics of *irx7* in embryonic retinas. (A–G) Dissected eyes obtained from embryos between 38 to 96 hpf. Anterior is to the left and dorsal is up. The black arrowheads indicate the *irx7+* cells (blue colour) in the retina, the dashed lines indicate the choroid fissure, while the red arrowheads in (D and E) indicate the posterior dorsal region of the retina, the last region to express *irx7*. (H) A schematic diagram of *irx7* expression dynamics in the retina from 38 to 52 hpf. The Roman numerals indicate the order of five retinal regions in which *irx7* appears sequentially. (I–L) Transverse retinal section of the corresponding whole-mount embryo at 38, 50, 72 and 96 hpf. Lateral is to the left and dorsal is up. The black arrowheads indicate the *irx7+* cells in the retina. Scale bars = 50 µm.

*Irx7*'s expression was first detected in a group of prospective INL cells in the anterior ventral retina at 38 hours post-fertilization (hpf) (Figure 1A and I, black arrowheads), when cells in the same region begin to withdraw from cell cycle [21]. Additional *irx7* expression was observed in the anterior dorsal retina (Figure 1B, black arrowhead) by 43 hpf, when most cells in the prospective INL have withdrawn from the cell cycle. At 46 hpf, *irx7* began to express in the posterior ventral retina (Figure 1C, black arrowhead). Then, its expression domain in both anterior and posterior retina gradually expanded to the dorsal side from 46 to 52 hpf (Figure 1C, D, E and J). At these stages, *irx7* seemed to be largely excluded from the basal INL region and was not expressed homogeneously in the remaining INL cells. When retinal lamination became apparent at 52 hpf, *irx7* appeared in the posterior dorsal retina, the last region to express *irx7* (compare Figure 1D and E, red arrowheads). By this stage, the initial expression wave of *irx7* in the prospective INL is completed. Since this wave overlaps with the cell cycle withdrawal and cell differentiation in INL, it is possible that *irx7* regulates these processes.

**Figure 2:**
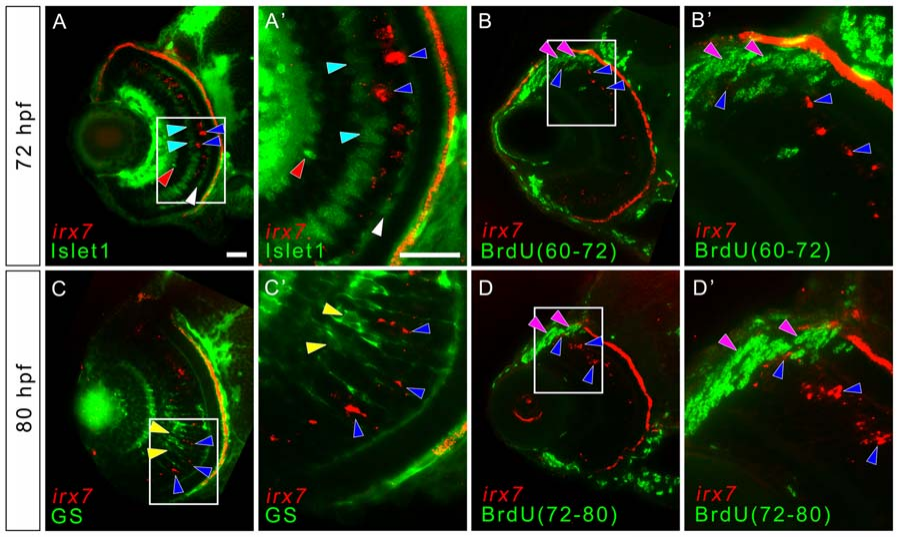
*Irx7* expresses in non-proliferative cells that are likely to be undifferentiated precursors in mature retina. To detect for the co-localization of *irx7* and ACs, BCs, HCs, MCs and proliferative cells in the more differentiated part of the WT retina, *in situ* hybridization of *irx7* was conducted in conjunction with immunostaining of anti-Islet1 for ACs, BCs and HCs, anti-GS for MCs and anti-BrdU for proliferative cells. (A) *irx7 in situ* hybridization with anti-Islet1 immunostaining at 72 hpf. The blue arrowheads indicate the *irx7+* cells (red colour) in the retina, while the red, cyan and white arrowheads indicate ACs, BCs and HCs respectively (all in green colour). (A′) The magnified view of the white box in (A). (B and D) The retina of embryos treated with BrdU from 60 to 72 hpf and 72 to 80 hpf respectively. The blue arrowheads indicate *irx7+* cells (red colour), while the pink arrowheads indicate BrdU+ cells (green colour). (B′ and D′) The magnified view of the white box in (B and D) respectively. (C) *irx7 in situ* hybridization with anti-GS immunostaining at 80 hpf. The blue arrowheads indicate the *irx7+* cells (red colour), while the yellow arrowheads indicate MCs (green colour). (C′) The magnified view of the white box in (C). Lateral is to the left and dorsal is up for all sections. Note that the RPE layer also showed red fluorescence but that was not a real signal. It was an artifact of the pigmentation in RPE. Since the images of the *in situ* hybridization were inverted before combining with the fluorescent images obtained from the immunostaining, the darker pigment in RPE, as well as the intense *in situ* colour, would appear as signal in this transformation. Scale bars = 20 µm.

Then, *irx7* expression was gradually restricted to a small group of cells in the outer INL at 72 hpf (Figure 1F and K) and was weakly detected in a few cells at 96 hpf (Figure 1G and L). These cells were located next to the HCs, which have a distinctive flattened morphology, and thus they might be a subset of BCs or MCs. Nonetheless, these *irx7+* cells rarely co-localized with ACs, BCs and HCs labeled by anti-Islet1 (N = 7) (Figure 2A and A′), and MCs labeled by anti-GS (N = 4) (Figure 2C and C′). There was also no overlap between the *irx7+* and proliferative cells that were undergoing S-phase from 60 to 72 hpf (N = 6) and 72 to 80 hpf (N = 6) (Figure 2B, B′, D and D′). Thus, after its initial expression wave at 52 hpf, *irx7* is expressed in a small group of the post-mitotic INL precursors or in a subtype of BCs that are not labeled by anti-Islet1, but it is not likely to express in MCs.

### Specific knockdown of Irx7

**Figure 3:**
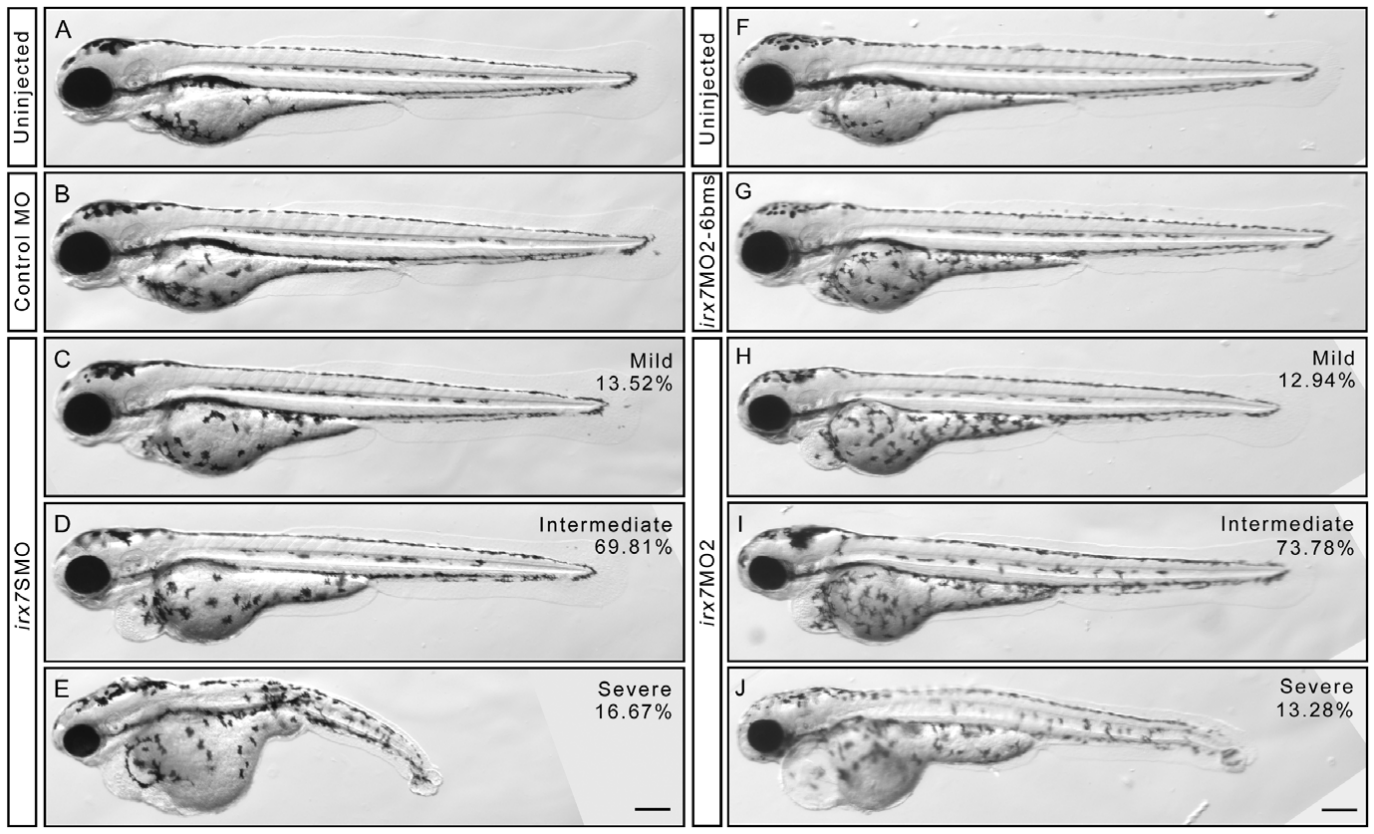
The phenotypes of *irx7*SMO and *irx7*MO2 morphants at 72 hpf. Phenotypic analysis of the optimized *irx7*SMO (A–E) and *irx7*MO2 (F–J) injection experiments. (A) The lateral view of an uninjected control embryo for *irx7*SMO injection experiments. (B) An embryo injected with 10 ng of Control MO. (C–E) Three phenotypic categories of the morphants after injected with 10 ng of *irx7*SMO. The percentage of embryos that had mild (C), intermediate (D) and severe (E) phenotypes was 13.52% (N = 106), 69.81% (N = 444) and 16.67% (N = 86) respectively. (F) The lateral view of an uninjected control embryo for *irx7*MO2 injection experiments. (G) An embryo injected with 3 ng of *irx7*MO2-6 bms. (H–J) Three phenotypic categories of morphants after injected with 3 ng of *irx7*MO2. The percentage of embryos that had mild (H), intermediate (I), and severe (J) phenotypes were 13.28% (N = 38), 73.78% (N = 211) and 12.94% (N = 37) respectively. Scale bar = 200 µm.

**Figure 4:**
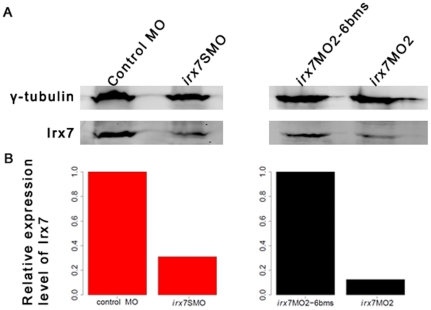
Microinjection of *irx7*SMO (10 ng) and *irx7*MO2 (3 ng) drastically reduces Irx7 protein level. The heads of 35 embryos injected with control MO and *irx7*SMO, and 30 embryos injected with *irx7*MO2-6 bms and *irx7*MO2 were dissected at 72 hpf. Proteins were then extracted from these samples and Irx7 expression detected by Western blot using anti-Irx7-234 (A). Γ-tubulin was used as a loading control. The specificity of the Irx7 antibodies was first confirmed by Western blot using various recombinant Irx7 proteins of different lengths (Table S1 and File S3) expressed from bacterial culture (data not shown). Using the information extracted from the Western blot, the protein level of Irx7 was found to be reduced by 69.01% and 87.58% respectively in the dissected heads of *irx7*SMO and *irx7*MO2 morphants when compared to the corresponding controls (B).

To study the roles of *irx7* during retinal development, Irx7 in developing embryos was knocked-down by microinjection of morpholinos (MOs). One splice-blocking MO (*irx7*SMO) [13] and two translation-blocking MOs (*irx7*MO1 and *irx7*MO2) [10], [12] were tested. Since the injection of *irx7*MO1's mismatch control, *irx7*MO1-5bms, always led to severe developmental defects (data not shown), this set of MOs was not used for further characterization. For the remaining two MOs, 10 ng of *irx7*SMO and 3 ng of *irx7*MO2 were chosen for further characterization of the retinal phenotype. The usage of these amounts was supported by the following three lines of evidence: First, the injection of these amounts led to an equivalent distribution of the resulting phenotypic categories in the two groups of MO-injected embryos (morphants), while controls did not show obvious developmental defects (Figure 3). Specifically, there were three phenotypic categories: mild, intermediate and severe (*irx7*SMO: N = 106, 444 and 86; *irx7*MO2: N = 38, 211 and 37 respectively) and there was no difference in their distribution (chi-squared = 1.93, df = 2, *p-*value = 0.38). Besides, the intermediate category was also the most frequent in both types of morphants (*irx7*SMO: 69.8% (444/636); *irx7*MO2: 73.8% (211/286)), and was further analyzed in the following experiments except for *in situ* hybridization (see below). Second, Irx7 protein from microdissected heads of *irx7*SMO (N = 35) and *irx7*MO2 (N = 30) morphants was reduced by 69.01% and 87.58% respectively compared with controls at 3 days post-fertilization (dpf) (Figure 4). Together with the specific effect on eye size and development that will be discussed below, these results strongly indicate that the microinjection of the optimized amount of MOs can specifically knock down Irx7 in developing zebrafish retinas. The immunostaining results of the knockdowns obtained from both MOs were very consistent and those obtained from *irx7*SMO morphants are discussed below.

### Irx7 knockdown reduces eye size and compromises retinal lamination

**Figure 5:**
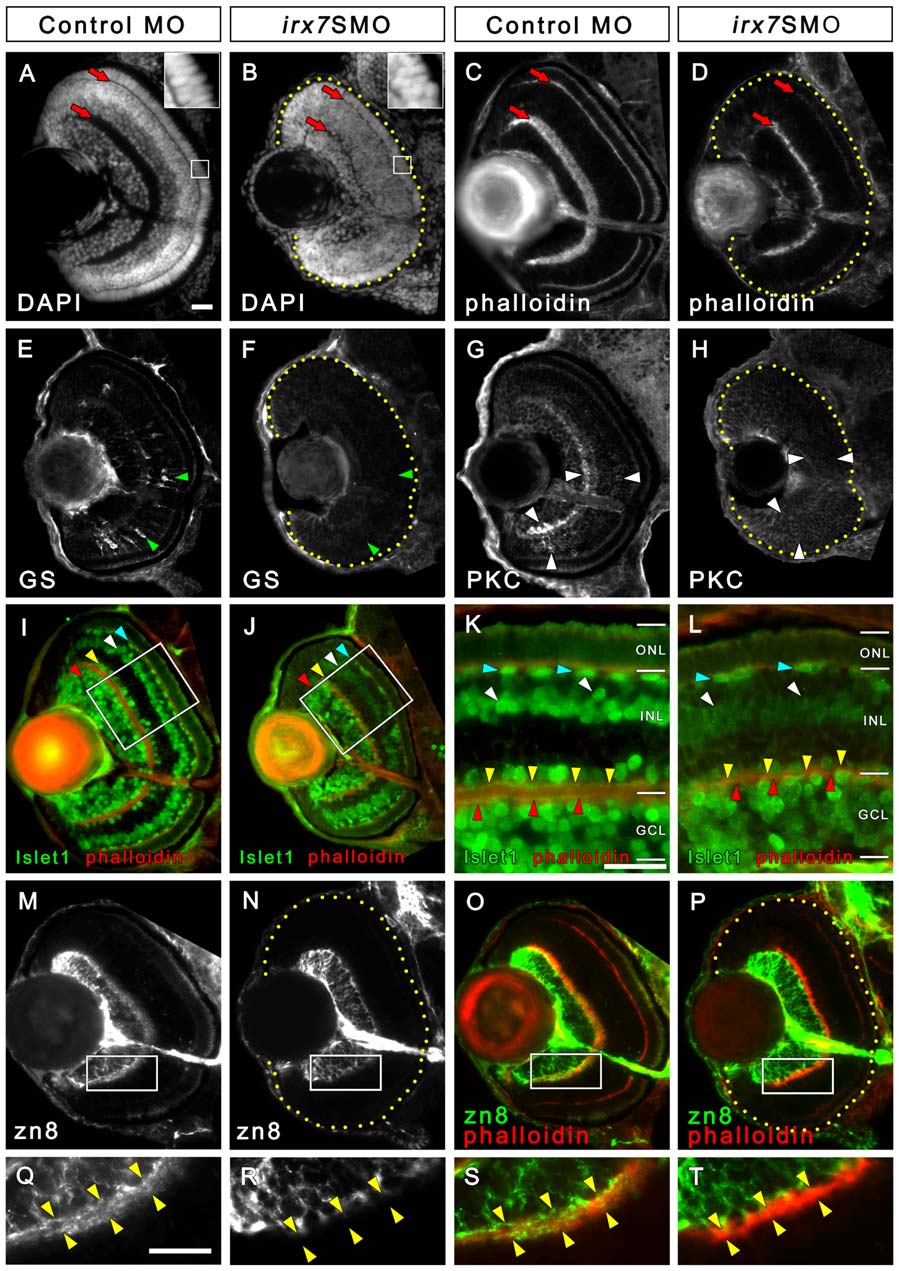
Irx7 knockdown reduces eye size and compromises retinal lamination, INL cells differentiation and dendritic projection of GCs into the INL. Irx7 knockdown reduced eye size and compromised retinal lamination at 72 hpf, as indicated by the DAPI (A and B) and phalloidin (C and D) stains that highlight nuclei and plexiform layers respectively. The red arrows indicate the IPL and OPL. The insets of (A) and (B) also show that the normal elongated morphology of the photoreceptors in control was compromised in the Irx7 morphant. The INL cells differentiation was analyzed by anti-GS for MCs (E and F), anti-PKC for BCs (G and H) and anti-Islet1 for ACs, BCs and HCs (I–L) at the same stage. Irx7 knockdown did not decrease the zn8+ GCs (N) compared with the controls (M), except for the elimination of a fuzzy domain on the apical side of the GCL (compare Q and R). This domain likely represents the dendritic projections of the GCs into the IPL, as it overlapped with the phalloidin staining of the IPL substantially (O and S). This overlap was completely absent in the morphants (P and T). Lateral is to the left and dorsal is up for all sections, except for (K and L), in which the apical side of retina is up. In addition, the retinal region in the samples with weak signal is highlighted by a dotted yellow line. The features indicated by the arrowheads are further discussed in the text. Scale bars = 20 µm.

The eye size of *irx7*SMO and *irx7*MO2 morphants was reduced, as shown by cryosectioning at 72 hpf (Figure 5A–D, also see Figure 3). The retinal area was measured from sections obtained from ten independent experiments. For each section, attempts were made to cut through the optic nerves in both eyes to maximize comparability across sections. There were five conditions in these experiments: *irx7*SMO (N = 70), control MO (N = 69), *irx7*MO2 (N = 59) and *irx7*MO2-6 bms (N = 63) morphants, and uninjected embryos (N = 49). Out of 310 sections analyzed, 195 contained optic nerves in both eyes and the remaining 115 contained a complete optic nerve in one eye. To delineate the specific effect on retinal area by Irx7 knockdown and to differentiate that from potential confounding effects caused by a difference in sectioning plane (i.e. with optic nerve in one vs. both eyes), and intrinsic variation of embryo size collected from different experiments, a linear mixed-effects model was fit (File S1). The results indicate that there was a specific and consistent decrease in retinal area in the morphants compared with controls (*irx7*SMO vs. control MO (ratio of area from the fitted model ± standard error): 72.92±5.81%; *irx7*MO2 vs. *irx7*MO2-6 bms: 71.54±6.37%). This further supports that the optimized MO injection amounts would reveal specific knockdown effect on retinal development.

The retinal cells in the morphants appeared abnormal compared with the controls (Figure 5A and B). For example, the INL was not stained as an intense apical sub-layer and a less intense basal sub-layer. Some INL cells appeared to be elongated along the radial axis, while photoreceptors appeared less elongated (Figure 5A and B, insets). Moreover, both IPL and OPL were compromised and appeared thinner (Figure 5A–D, red arrows). To determine the extent of the thinning of the INL, its thickness was measured from sections collected from three independent experiments (total N = 136 for each of the morphant and control group). To facilitate comparison between sections and experimental conditions, the thicknesses of INL immediately dorsal and ventral to the optic nerve was measured. A linear-mixed effect model was fit for each thickness measurement. The results indicate that the INL thickness was reduced in *irx7*SMO morphant (*p*-values<0.0001 for both dorsal and ventral INL thickness). These results suggest that Irx7 knockdown might affect retinal differentiation and/or neurite outgrowth into the plexiform layers.

### Irx7's function is essential for INL cells differentiation

To investigate the effect of Irx7 knockdown on INL cells differentiation, immunostaining was conducted with cell-type specific markers for MCs, ACs, BCs and HCs at 72 hpf. Zebrafish shows the first visual response at this stage [22], hence all INL cell types should be present. First, MCs was absent in the morphants, as indicated by a lack of anti-GS staining (Figure 5E and F, green arrowheads) (File S1; multiple comparison test after Kruskal-Wallis, adjusted *p*-value<0.05). Also, Irx7 knockdown eliminated the GFP signal in the INL of *Tg*(*gfap:GFP*)*^mi2001^* [23], a line that labels MCs, compared with the controls at 59 hpf (Figure S1A and B). At 72 hpf, the number of GFP+ MCs per retinal area was still lower in the morphants compared with the controls (File S1; multiple comparison test after Kruskal-Wallis, adjusted *p*-value<0.05). Further, some GFP+ MCs in the morphants were mis-located to the apical INL and their radial processes were mostly absent (Figure S1C and D). These analyses indicate that MCs differentiation was compromised by Irx7 knockdown.

Second, BCs differentiation was investigated by anti-PKC and anti-Islet1 stainings. At 72 hpf, much of the PKC+ signal was seen in the IPL in the controls. This is presumably originated from the BCs projections (Figure 5G, white arrowheads). Also, some BCs were detected on the ventral side of the retina and in the middle INL. These stainings were largely absent in the morphants (Figure 5H) and the count distribution of PKC+ staining patterns between the morphants and controls was different (File S1; multiple comparison test after Kruskal-Wallis, adjusted *p*-value<0.05). In addition, Islet1+ signal was detected around the BCs region in both morphants and controls, but the staining was less intense and the BCs was slightly more elongated in the morphants compared with the controls (Figure 5I–L, white arrowheads). Together, these data suggest that at least a subset of BCs in the morphants were not fully differentiated.

The anti-Islet1 staining also revealed issues with ACs and HCs differentiation (Figure 5I–L, yellow and cyan arrowheads respectively). First, the number of ACs per retinal area in the morphants was reduced compared with the controls (File S1; two-tailed Student's *t*-test, *p*-value = 0.021). In addition, many of these Islet1+ ACs in the controls could extend neuronal projections into a single IPL lamina (Figure 5K, yellow arrowheads), while this was rarely observed in the morphants (Figure 5L, yellow arrowheads). Indeed, only two out of 16 ACs in one morphant showed signs of neuronal projections. As a result, there were more ACs with IPL projections in control retinas (median (*M*) = 43.1%, median absolute deviation (MAD) = 7.4%, N = 6) than that in the morphants (*M* = 0%, MAD = 0%, N = 7) (File S1; logistic regression; *p-*value = 1.33e-05). For HCs, there were fewer Islet1+ HCs per retinal area in the morphants compared with the controls (File S1; two-tailed Welch two sample *t*-test, *p*-value = 0.00056). Together, these results indicate that the differentiation of all four INL cell types is compromised in the Irx7-deficient retinas.

### Irx7's function is essential for photoreceptor differentiation and the dendritic outgrowth of GCs into the IPL

GCs in the morphants developed relatively well compared with other cell types, as indicated by anti-zn8 (Figure 5M–T) and anti-Islet1 (Figure 5I and J) stainings. For example, there was no difference in the number of zn8+ GCs per retinal area in the *irx7*SMO morphants (mean (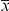) = 0.0071 µm^−2^, standard deviation (*s*) = 0.0012 µm^−2^, N = 9) compared with the controls (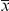 = 0.0068 µm^−2^, *s* = 0.00067 µm^−2^, N = 7) (File S1; two-tailed Welch two sample *t*-test, *t* = −0.6317, df = 13.06, *p*-value = 0.54). An optic nerve, which is composed of the GCs' axons, was formed in both conditions (Figure 5M and N). Nonetheless, the dendritic outgrowth of zn8+ GCs into the IPL was absent in the morphants (Figure 5R and T, yellow arrowheads; compared to the normal projections in controls in Figure 5Q and S). This outgrowth issue was also observed in Islet1+ GCs (Figure 5K and L, red arrowheads), in which the projection into a single lamina was entirely absent in the morphants.

**Figure 6:**
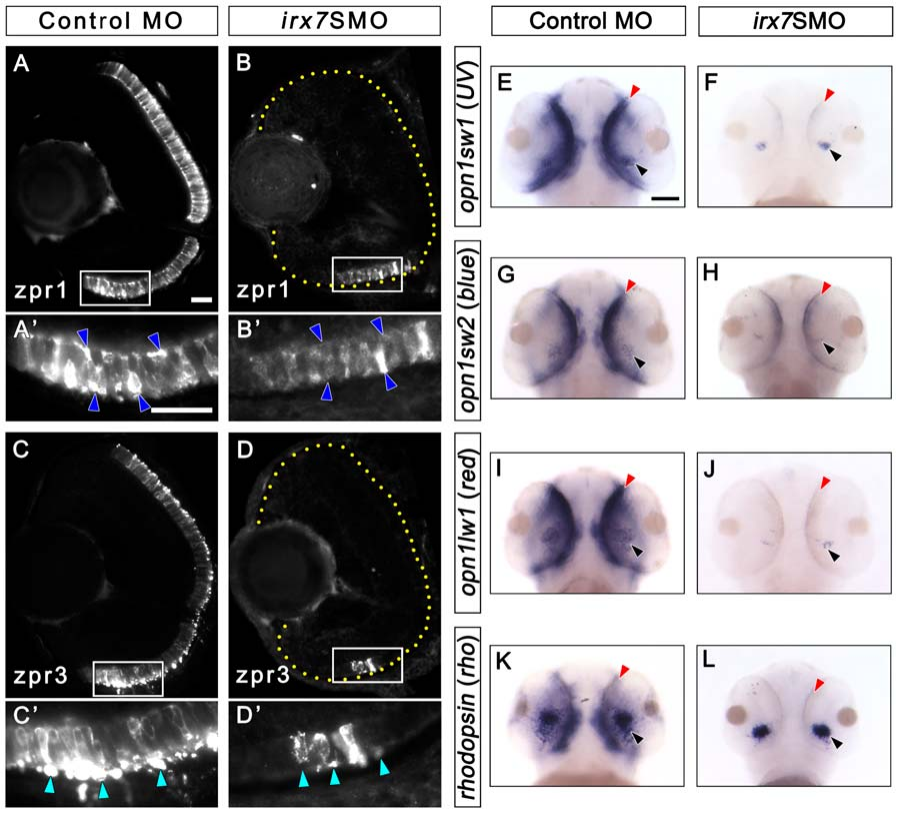
Irx7 knockdown compromises photoreceptor differentiation at 72 hpf. Irx7 knockdown compromised the staining of anti-zpr1 for red-green double cones and anti-zpr3 for rods in the morphants (B and D) compared with the controls at 72 hpf (A and C). (A′, B′, C′ and D′) The corresponding magnified view of the positive signal area in the white box in A, B, C and D. The features indicated by the arrowheads are further discussed in the text. Lateral is to the left and dorsal is up for all sections. In addition, the retinal region in the samples with weak fluorescent signal is highlighted by a dotted yellow line. (E–L) Whole-mount *in situ* hybridization of three cone *opsins* (*uv*, *blue* and *red*) and one rod *opsin* (*rho*) also indicate the differentiation of these photoreceptors was compromised. The most common staining pattern is shown. The black arrowheads indicate the specific staining (blue colour) of the ventral patch, while the red arrowheads indicate the staining in the ONL. Embryos were imaged from the ventral side and anterior is up. Scale bars = 20 µm for (A)–(D) and 50 µm for (E)–(L).

Despite *irx7* is only expressed in the INL, its knockdown also affected the ONL (Figure 5A and B, insets). This was further investigated by immunostaining with anti-zpr1 and anti-zpr3 for red-green double cones and rods respectively at 72 hpf (Figure 6). In controls, zpr1+ cells were detected in the whole ONL (Figure 6A), while most morphants had a few zpr1+ cells in the ventral ONL (Figure 6B) or there was no positive signal at all. (File S1; two-tailed Wilcoxon rank sum test, *p*-value = 0.0034). In addition, the staining was intense on both the apical and basal sides of the zpr1+ cells in the controls (Figure 6A′, blue arrowheads), but this was not apparent in the morphants (Figure 6B′, blue arrowheads). A similar pattern was observed for anti-zpr3, in which zpr3+ cells were detected in the entire ONL in the controls (Figure 6C), while it was only detected in a small group of cells in the ventral ONL in most morphants (Figure 6D) (File S1; two-tailed Wilcoxon rank sum test, *p*-value = 0.00058). Also, the intense zpr3 staining in the outer segments of rods in the controls (Figure 6C′, cyan arrowheads) was not observed in the morphants (Figure 6D′, cyan arrowheads).

To further characterize the effect of Irx7 knockdown on photoreceptors, *in situ* hybridization of three opsins (*opn1sw1*: *UV*, *opn1sw2*: *blue* and *opn1lw1*: *red*) for three cone types and *rho* for rods was performed at 72 hpf. Since it was more difficult to differentiate the phenotypic categories after treating the morphants with PTU (phenylthiourea), all embryos were used in this type of *in situ* hybridization analysis (File S1). First, all opsins were generally widely expressed in the controls (Figure 6E, G, I and K; an embryo from the most common category is shown). In particular, they were all expressed in the ventral patch and extensively in the ONL; while in the morphants, all *opsins* were expressed at a weaker level in the ventral patch, especially for *opn1lw1* (Figure 6F, H, J and L). Also, these opsins were almost not expressed in the ONL except for *opn1sw2*, which was weakly expressed in the ONL. The *in situ* hybridization pattern of each gene was artificially classified into different categories and the number of embryos in different categories counted by the experimenter and a blind observer (File S1 and Table S2). A similar approach has been previously used to analyze photoreceptor differentiation defects in zebrafish development [24]. The results between the two observers were highly concordant and the data from the experimenter are presented. The count distribution of the staining patterns in morphants and controls was different for all *opsins* analyzed (File S1; Bonferroni-adjusted *p-*value = 6.48e-05, 0.013, 6.04e-6 and 0.021 for *opn1sw1*, *opn1sw2*, *opn1lw1* and *rho* respectively). Thus, despite *opsins* expression is somewhat affected in the controls compared with the uninjected embryos (Bonferroni-adjusted *p-*value<0.05 in all cases), Irx7 knockdown has specifically decreased *opsins* expression even further. Taken together, these results suggest that despite *irx7* only expresses in the INL, its function is essential for photoreceptor differentiation and the dendritic outgrowth of GCs into the IPL.

A delay in cell cycle withdrawal and compromise of plexiform layers, but not apoptosis, may contribute to the eye size reduction in the Irx7 morphants.

**Figure 7:**
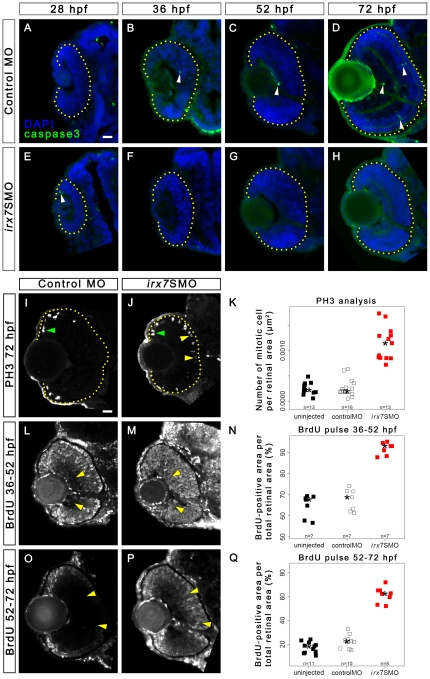
Irx7 knockdown does not induce apoptosis but can delay cell cycle withdrawal. Immunostaining of embryos injected with 10 ng of control MO or *irx7*SMO were conducted with anti-active caspase3 antibody (green colour) at 28, 36, 52 and 72 hpf. The nuclei were counterstained with DAPI (blue colour). In both controls (A–D) and *irx7*SMO morphants (E–H), only a few active caspase3+ cells (white arrowheads) were detected in some retinas at all stages. All cells that showed a positive anti-active caspase3 signal had the characteristic cell shrinkage and rounded morphology, as shown by the DAPI staining. While all active caspase3- cells looked healthy. Mitotic cells were detected by anti-PH3 in the retinas of controls (I) and morphants (J) at 72 hpf. PH3+ cells in the MZ and in the ectopic apical retina are indicated by green and yellow arrowheads respectively. (K) A stripchart of the number of PH3+ cell per retinal area in uninjected embryos, controls and morphants. Retinal cells that had gone through S-phase in controls and morphants were also detected by BrdU incorporation from 36 to 52 hpf (L and M) and from 52 to 72 hpf (O and P). (N and Q) The corresponding stripcharts of BrdU+ area per retinal area in the uninjected embryos, controls and morphants. The asterisks in all stripcharts represent the median of each group. Lateral is to the left and dorsal is up for all sections. The retinal region in these samples is highlighted by a dotted yellow line. Scale bar = 20 µm.

To address the cause of the eye size reduction in the morphants, cell cycle and apoptotic statuses were assessed. To detect apoptotic cells, immunostaining of anti-active caspase3 was conducted at 28, 36, 52 and 72 hpf (Figure 7). All retinas at 28, 36 and 52 hpf had fewer than five active-capsase3+ cells per retina, and there was no difference in these numbers between all conditions at these stages (data not shown). At 72 hpf, there was an increase in the number of active-caspase3+ cells in the controls compared with morphants (Figure 7D and H; File S1; multiple comparison test after Kruskal-Wallis, adjusted *p-*value<0.05), but there was no difference between the uninjected embryos and controls or morphants (adjusted *p-*value>0.05 in both cases). While the results show that control MO injection slightly induced apoptosis at 72 hpf, they argue against the role of apoptosis in eye size reduction of morphants.

To investigate the potential change in the cell cycle status, immunostaining was conducted on cryosections using an M-phase (anti-PH3) and an S-phase marker (anti-BrdU). The former analysis was conducted on samples collected at 28, 36, 52 and 72 hpf. There was no difference in the number of PH3+ cells per retinal area as well as the distribution of the staining pattern counts between morphants and controls at 28, 36 and 52 hpf (data not shown). At 72 hpf, PH3+ cells were only detected in the proliferative marginal zone (MZ) in control retinas (Figure 7I, green arrowhead). While in *irx7*SMO morphants, PH3+ cells were detected in both MZ (Figure 7J, green arrowhead) and on the apical side of retina (Figure 7J, yellow arrowheads), an ectopic location for these cells at this stage. There was an increase in the number of PH3+ cells per retinal area in the morphants compared with controls and uninjected embryos (Figure 7K and File S1; multiple comparison test after Kruskal-Wallis, adjusted *p-*value<0.05 in both comparisons), but not among the latter two groups (adjusted *p-*value>0.05). To detect cells that had gone through the S-phase of the cell cycle from 36 to 52 hpf and from 52 to 72 hpf, embryos were treated with 10 mM BrdU during these periods and the labeled cells detected by anti-BrdU immunostaining. These two periods cover the approximate stage when the progenitors in the prospective INL and ONL withdraw from the cell cycle [21], and the initial wave of *irx7* expression in the retina (Figure 1). In the morphants, most progenitors in the prospective GCL and INL in the 36–52 hpf group (Figure 7M, yellow arrowheads), and many of them in the INL and ONL in the 52–72 hpf group (Figure 7P, yellow arrowheads) were BrdU+. While in the corresponding controls, cells in these areas were primarily BrdU- (Figure 7L and O, yellow arrowheads). As a result, the BrdU+ area per total retinal area, as traced in the cryosections, was evidently higher in the *irx7*SMO morphants (Figure 7N and Q). Since many cells in the GCL and INL in the morphants became BrdU- in the 52 to 72 hpf group ultimately (Figure 7P), together with the over-abundance of PH3+ cells in the morphant retinas at 72 hpf (Figure 7J), these results suggest that the lack of a functional Irx7 in developing retinas delays but not completely blocks cell cycle withdrawal.

### Irx7 regulates the expression of TFs that specify INL cells

**Figure 8:**
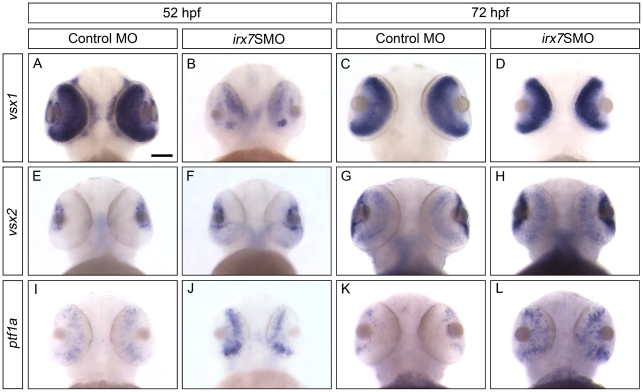
Irx7 regulates the expression of TFs that specify retinal cell types in INL at 52 and 72 hpf. Whole-mount *in situ* hybridization of *vsx1* (A–D), *vsx2* (E–H), *ptf1a* (I–L) was conducted. The most common staining pattern is shown. Embryos were imaged from the ventral side and anterior is up. See text for further discussions. The results for *neurod*, a TF that specifies cells in both INL and ONL, will be presented in Figure 9. Scale bar = 50 µm.

**Figure 9:**
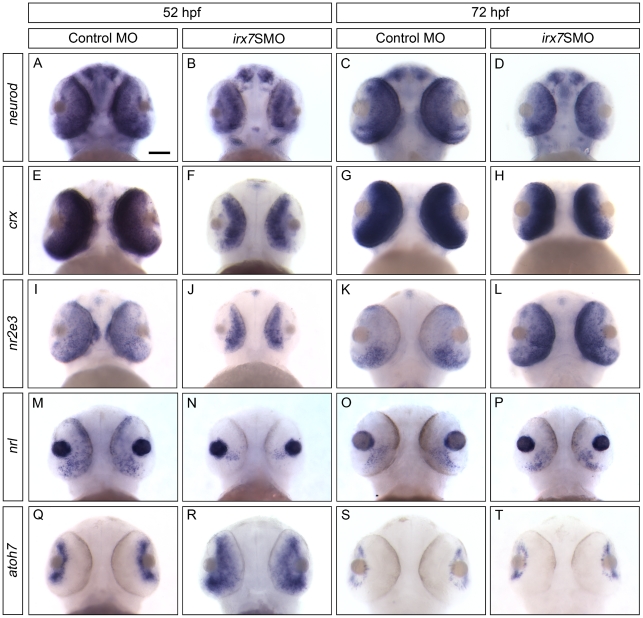
Irx7 regulates the expression of TFs that specify retinal cell types in ONL and GCL at 52 and 72 hpf. Whole-mount *in situ* hybridization of *neurod* (A–D), *crx* (E–H), *nr2e3* (I–L), *nrl* (M–P) and *atoh7* (Q–T) was conducted. The most common staining pattern is shown. Embryos were imaged from the ventral side and anterior is up. See text for further discussions. Scale bar = 50 µm.

Irx7's effects on retinal differentiation and lamination could be mediated through downstream TFs that specify different retinal cell types. To determine these downstream targets in the INL, *in situ* hybridization was performed at 52 and 72 hpf, using probes for genes that can specify different retinal cell types (Figure 8 and Figure 9; see File S3 for literature references for these probes). These include *vsx1* and *vsx2* for two distinct sub-populations of BCs, *ptf1a* for ACs and HCs, *neurod* and *vsx1* for ACs and *vsx2* for MCs. All phenotypic categories of the knockdown embryos were used in the analysis. For each gene, there would be a unique range of staining patterns (Table S2), which were counted and analyzed as in the case of *opsins* (File S1). All comparisons for the count distribution of staining patterns of morphants and controls discussed below were significant (Bonferroni-adjusted *p*-value<0.05). The most representative pattern is shown in the figures. In addition, the regulations of Irx7 on these genes are summarized in Table 1 and discussed below.

**Table 1:** The change in the expression level and/or pattern of target genes in Irx7 knockdown experiments.

Gene Name	Stage	Expression pattern in control MO morphants	Chang in expression level and/or pattern in representative *irx7*SMO morphants	Inferred regulation by *irx7* in WT embryos
*atoh7*	52 hpf	MZ	Higher & wider in neural retina	Negative
*atoh7*	72 hpf	MZ	Higher	Negative
*crx*	52 hpf	ONL	Lower	Positive
		INL	Higher	Negative
*crx*	72 hpf	ONL	Lower	Positive
		INL	Higher	Negative
*neurod*	52 hpf	ONL	Lower	Positive
		INL	Higher	Negative
*neurod*	72 hpf	ONL	Lower	Positive
		INL	Higher	Negative
*nr2e3*	52 hpf	ONL	Lower	Positive
		INL	Higher	Negative
*nr2e3*	72 hpf	ONL	Higher	Negative
*nrl*	52 hpf	ONL	Lower	Positive
*nrl*	72 hpf	ONL	Higher	Negative
*opn1sw1 (uv)*	72 hpf	Ventral patch & ONL	Lower	Positive
*opn1sw2 (blue)*	72 hpf	Ventral patch & ONL	Lower	Positive
*opn1lw1 (red)*	72 hpf	Ventral patch & ONL	Lower	Positive
*ptf1a*	52 hpf	INL	Higher	Negative
*ptf1a*	72 hpf	MZ	Higher in INL	Negative
*rho*	72 hpf	Ventral patch & ONL	Lower	Positive
*vsx1*	52 hpf	INL	Lower	Positive
*vsx1*	72 hpf	INL	Higher	Negative
*vsx2*	52 hpf	MZ	Higher	Negative
*vsx2*	72 hpf	MZ	Higher	Negative
		INL	Higher	Negative

The expression level and/or pattern of the candidate target genes were determined from representative *irx7*SMO morphants and controls. The resulting regulatory relationship between Irx7 and these candidate genes was also inferred by comparing these two sample groups. See Figure 11 for the resulting network, Figure S2 for the comprehensive network that also includes additional connections of the candidate genes as extracted from literature references (File S2).

*Vsx1* was widely expressed in the INL in the controls at both 52 (Figure 8A) and 72 hpf (Figure 8C), its expression at 72 hpf was also slightly more intense than the uninjected embryos (data not shown). While its expression was down-regulated in the morphants at 52 hpf (Figure 8B), it was slightly up-regulated by 72 hpf (Figure 8D). *Vsx2* was expressed in the MZ at 52 (Figure 8E) and 72 hpf (Figure 8G) in the controls, while it was also expressed at a weaker level in the INL at 72 hpf (Figure 8G). In the morphants, *vsx2* showed a slightly stronger expression in the MZ and the ventral temporal retina compared with the controls at 52 hpf (Figure 8F). By 72 hpf, its expression pattern was similar to that in the controls, except it was still more intense in the INL and MZ (Figure 8H). *Ptf1a* was expressed in the prospective ACs and HCs in the INL of the controls at 52 hpf (Figure 8I), while its expression was restricted to the MZ by 72 hpf (Figure 8K). Its expression at 52 hpf was also slightly stronger than the uninjected embryos (data not shown). In the morphants, *ptf1a*'s expression was consistently up-regulated at both 52 (Figure 8J) and 72 hpf (Figure 8L); and its up-regulation at 72 hpf originated from an ectopic expression in the INL. *Neurod* was primarily expressed in the prospective AC region in the INL at both 52 (Figure 9A and data not shown) and 72 hpf (Figure 9C and data not shown) in the controls. In the morphants, *neurod* expression in the INL was more intense and widespread at 52 hpf (Figure 9B and data not shown); while its expression became more restricted to the AC region at 72 hpf but was still relatively widespread compared with the controls (Figure 9D and data not shown). Taken together, these results indicate that in the INL, *irx7* is essential for the transcriptional activation of *vsx1* at 52 hpf, and the transcriptional repression of *vsx1* and *vsx2* at 72 hpf, and *neurod* and *ptf1a* at both 52 and 72 hpf.

### Irx7 regulates the expression of TFs that specify photoreceptors and GCs

To identify the downstream TF targets of *irx7* that specify photoreceptors and GCs, a similar *in situ* hybridization analysis was conducted as in the case of INL. The specific gene probes used in this investigation include *neurod* and *crx* for rods and cones, *nr2e3* and *nrl* for rods, and *atoh7* for GCs. The regulations of Irx7 on these genes are summarized in Table 1 and are discussed below.

*Neurod* was intensely expressed in the ONL of controls at both 52 (Figure 9A and data not shown) and 72 hpf (Figure 9C and data not shown); while its expression was first suppressed in the ONL at 52 hpf (Figure 9B and data not shown) and became more widespread but not uniformly expressed at 72 hpf in the morphants (Figure 9D and data not shown). *Crx* was strongly expressed in the ONL and the outer INL in controls at 52 (Figure 9E and data not shown) and 72 hpf (Figure 9G and data not shown). Its expression in the morphants was strong in the inner INL and weak in the ONL except for the ventral patch at 52 hpf (Figure 9F and data not shown). By 72 hpf, the expression pattern of *crx* in these embryos became similar to that in the controls, but its expression level in the INL and ONL was slightly higher and lower comparatively (Figure 9H and data not shown). The expression of *nr2e3* was mainly restricted to ONL at 52 (Figure 9I and data not shown) and 72 hpf (Figure 9K and data not shown) in the controls, with an obvious decrease in the expression level and a restriction to the ONL in the ventral temporal retina at the latter stage (Figure 9K and data not shown). In the morphants, *nr2e3* expression was intense and more widespread in the INL, and was weaker in the ONL compared with the controls at 52 hpf (Figure 9J and data not shown). By 72 hpf, *nr2e3* expression became more restricted to the ONL with an obvious ventral patch expression, and its expression level was substantially higher than the controls (Figure 9L and data not shown). There was still ectopic *nr2e3* expression in the INL of the morphants at this stage (data not shown). For *nrl*, it was intensely expressed in the controls' ONL, particularly in the posterior-ventral region at 52 hpf (Figure 9M), while it was also expressed in the dorsal ONL in the uninjected embryos (data not shown). In the morphants, the expression of *nrl* was suppressed except for a small ventral patch in the ONL at 52 hpf (Figure 9N). The expression pattern between the morphants and controls became more similar at 72 hpf (Figure 9O and P). In particular, it was expressed in a very restricted area on the posterior-ventral ONL. Nonetheless, *nrl* expression in the morphants was slightly higher at this stage. Finally, the expression pattern of *atoh7* was different between the morphants and controls at 52 hpf (Figure 9Q and R). In controls, *atoh7* expression was primarily restricted in the MZ, while it had a wider expression pattern in the retinas of many morphants in addition to the MZ. Also, the expression level of *atoh7* in the MZ of the morphants seemed to be higher than the controls. At 72 hpf, the expression of *atoh7* was similar between the morphants and controls (Figure 9S and T). While it was restricted to the MZ, the expression level remained slightly higher in many morphants. In short, *irx7*'s expression in the INL can ultimately activate the transcription of *neurod* and *crx* at both 52 and 72 hpf in the ONL, while it can activate *nr2e3* and *nrl* at 52 hpf but suppress them at 72 hpf. Further, *irx7*'s activity seems to be necessary for suppressing *atoh7* in the central retina at 52 hpf, and in the MZ at both 52 and 72 hpf.

### Irx7 is necessary but likely not sufficient for the development of INL and ONL

**Figure 10:**
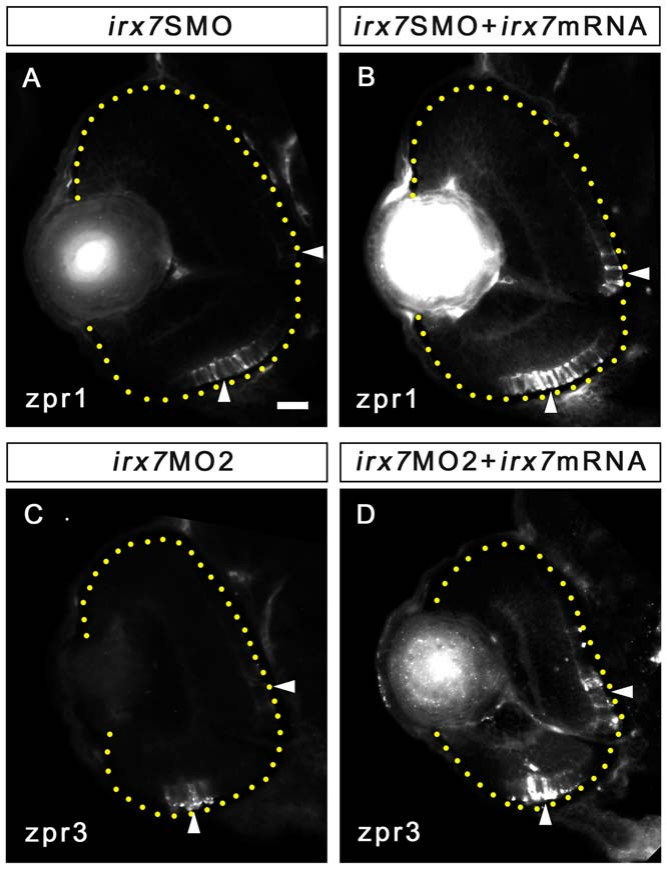
*Irx7* mRNA can partially rescue the effects caused by Irx7 knockdown. (A and B) Immunostaining of anti-zpr1 for cones on the retinal section of an morphant and a rescued embryo at 72 hpf respectively. (C and D) Immunostaining of anti-zpr3 for rods on the retinal section of an *irx7*MO2 morphant and a rescued embryo at 72 hpf respectively. Lateral is to the left and dorsal is up for all sections. The retinal region in the samples is highlighted by a dotted yellow line. The comparable regions in the retinas are highlighted by white arrowheads. Scale bar = 20 µm.

To rescue the phenotypes of *irx7*SMO and *irx7*MO2 morphants, five pgs of full-length *irx7* mRNA was co-injected with *irx7*SMO and *irx7*MO2 (See File S3 for optimization). First, there was a partial expansion in the staining domain of anti-zpr1 but not anti-zpr3 in rescued *irx7*SMO morphants. Specifically, in most *irx7*SMO morphants, zpr1+ signal was only detected in the ventral retina (Figure 10A); while in most of the corresponding rescued embryos such signal was detected not only in the ventral retina, but also in the central retina (Figure 10B). Despite the change in the pattern and seemingly large increase in the median of zpr1+ cells per retinal area in the rescued *irx7*SMO morphants (*M* = 0.0013 µm^−2^, MAD = 0.0012 µm^−2^, N = 10) compared with the *irx7*SMO morphants (*M* = 0.00021 µm^−2^, MAD = 0.00031 µm^−2^, N = 9), the increase in the zpr1+ cells was not different between the two groups (File S1; two-tailed Wilcoxon rank sum test, W = 25, *p-*value = 0.12). The main reason is that the phenotypic variation in the rescued embryos was large. Even though quite a few embryos had more zpr1+ cells in the central retina of the rescued group that was not observed in the morphant group, there were also a few embryos in the rescued group that number of zpr1+ cells was comparable to the morphant group; hence a lack of significance in this statistical test. Second, there was also an expansion in the staining domain of anti-zpr3 in the rescued *irx7*MO2 embryos (Figure 10D). In particular, additional staining was observed in the central retina compared to the corresponding *irx7*MO2 morphants (Figure 10C). The number of zpr3+ cells per retinal area in the rescued *irx7*MO2 morphants (*M* = 0.00083 µm^−2^, MAD = 0.00025 µm^−2^, N = 9) was higher compared with the *irx7*MO2 morphants (*M* = 0.00044 µm^−2^, MAD = 0.00015 µm^−2^, N = 9) (File S1; two-tailed Wilcoxon rank sum test, W = 16, *p-*value = 0.031). Also, there was no noticeable change of the immunostaining pattern for the markers that can recognize different cell types in the INL (data not shown). These data indicate that microinjecting 5pgs of *irx7* mRNA at one-cell stage can only partially rescue the effects caused by Irx7 knockdown at 72 hpf.

To determine if Irx7's activity is sufficient for driving the TFs that specify different cell types precociously, a slightly lower amount of *irx7* mRNA (3 or 4 pgs) that is not toxic to embryonic development or equal amount of eGFP were injected into wild-type (WT) embryos at one-cell stage. The expression of *atoh7*, *neurod*, *nr2e3*, *ptf1a* and *vsx1* was investigated by *in situ* hybridization at 52 hpf. There was no noticeable difference in the expression pattern and level of these TFs between the *irx7* mRNA and eGFP injected embryos (data not shown). Thus, the results indicate that over-expression of up to 4 pgs of *irx7* is likely not sufficient for driving the expression of these TFs precociously.

### Shha does not activate *irx7* in the INL

During zebrafish eye development, the expression of *shha* in GCs starting at 28 hpf is responsible for the propagation of neurogenic wave in the GCL [4]. A second, independent wave of *shha* expresses in the ACs starting at about 32 hpf [20] is essential for INL cells differentiation and retinal lamination. Since *irx7* starts to express in the INL at 38 hpf (Figure 1A) and Irx7-deficient retinas have defects in differentiation and lamination, it was hypothesized that Shha regulates these processes through activating *irx7* in the INL. To test this hypothesis, the Shha signal transduction was inhibited by treating the embryos with cyclopamine, a Shh inhibitor, starting at 24, 26, 30 and 36 hpf. The embryos were collected at 52 hpf and the *irx7* expression pattern in the retinas (regions I–V; as defined in Figure 1H) detected by *in situ* hybridization. The efficiency of the cyclopamine treatment on inhibiting Shha signaling pathway was confirmed by the suppression of *ptc1*, the receptor of Shh, with *in situ* hybridization [25]. The number of embryos with *irx7* expression up to a particular region in the cyclopamine-treated, ethanol (carrier)-treated and untreated embryos was counted. All treatment lengths yielded similar results in different trials, and the longest one, the 24–52 hpf group, is discussed here. In theory, this longest treatment would give the most drastic results because all known neurogenic waves mediated by Shha in the retina would have been sufficiently inhibited. The results showed that the count distribution of *irx7* expression patterns in these experimental groups was not different (File S1; two-tailed Fisher exact test, Bonferroni-adjusted *p-*value>0.5). Thus, the data do not support the hypothesis that Shha mediates its effects on retinal development through activating *irx7* in INL.

## Discussion

### The expression of Irx7 in the prospective INL is essential for INL cells differentiation and retinal lamination

**Figure 11:**
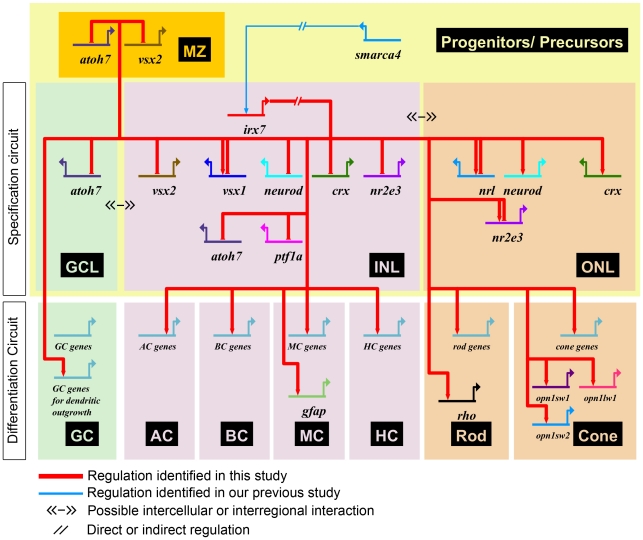
Irx7 gene regulatory network for zebrafish retinal development. A gene regulatory network was constructed using the expression patterns of *irx7* downstream targets as characterized in this study. The specification circuit of the network consists of TFs that specify different retinal cell types while the differentiation circuit consists of genes that carry out cell type specific functions. For example, *opsins* in the photoreceptors are responsible for visual signal transduction. Genes that have not been fully characterized yet are represented by a generic gene (*cell type*-*genes*) in the differentiation circuit. The activation of these “*cell type*-genes” by *irx7*, as well as by other TFs, symbolizes the differentiation of the corresponding cell types driven by the specification circuit. For GCs, an additional *“GC genes for dendritic outgrowth”* is created to distinguish the specific effects of *irx7* knockdown on their dendritic outgrowth (Figure 5). If the actual location of the interaction is not well defined, the domain/cell type in which the effector gene is expressed will be used. In addition, different retinal regions, including, GCL, INL and ONL can have cellular interaction (≪-≫) that can trigger signal transduction and in turn modulate gene expression. Note that the network topology is a static global view which consists of information obtained from different stages and studies, thus some genes may have both positive and negative inputs if the regulation is dynamical during development. See File S2 for supporting evidence of the connections and Figure S2 for a comprehensive network with additional connections between these genes as extracted from other literature references.

This study has revealed several key roles of Irx7 in the INL development. First, the commencement of *irx7* expression in the prospective INL cells coincides with their cell cycle withdrawal [21]. Together with *irx7's* extensive expression in the INL (Figure 1), they suggest that Irx7 is potentially involved in INL cells differentiation and retinal lamination. Indeed, Irx7 knockdown has compromised both processes (Figure 5). Irx7 likely controls retinal differentiation by transcriptionally regulating known TFs that specify various retinal cell types (Figure 8 and Figure 9). This specification circuit (Figure 11, also see a comprehensive circuit with additional connections from literature in Figure S2) will in turn regulate specific genes in the differentiation circuit that mediate essential functions in the corresponding differentiated cells, including the neuronal projections into the plexiform layers (Figure 5A–D, K and L). Alternatively, Irx7 may drive the genes in this differentiation circuit directly, but this is not determined by the current experimental design. Thus, the results from this study are more consistent with the hypothesis that Irx7 controls retinal lamination through regulating INL cells differentiation.

How does the exclusive expression of *irx7* in the INL mediate retinal lamination? The results from this study suggest a possibility that this is through the regulation of the differentiation of INL cells. In Irx7-deficient retinas, the neuronal projections from ACs (Figure 5K and L), BCs (Figure 5G and H) and GCs (Figure 5K–T and data not shown) into the IPL were eliminated or reduced, and this in turn compromised the IPL (Figure 5A–D). Interestingly, GCs differentiation was not severely compromised (Figure 5M–T) despite a slight cell cycle withdrawal delay (Figure 7) and an early alteration of *atoh7* at 52 hpf but not 72 hpf in the central retina (Figure 9). This is likely because most prospective GCs have already withdrawn from cell cycle by the stage when *irx7* first expresses in the prospective INL at 38 hpf (Figure 1) [21]. The lack of GCs' dendritic projections into the IPL is likely a consequence of the differentiation issues in the INL. MCs are not likely a mediator for lamination even though their differentiation is also compromised (Figure 5E and F) and they have rudimentary processes in the IPL at 55 hpf [26]. This is because they are generally the last cell type to differentiate in the retina and that precludes them as a prime driver of retinal lamination, which begins at an earlier stage. Indeed, Williams et al. have demonstrated that MCs also do not play a role in the formation of outer retinal synapses, further supporting this idea. Among the remaining two cell types that project to the IPL, BCs are also a late cell type. Thus ACs, being an early cell type, are the likely candidate that drives the formation of IPL.

Research from others has indicated that ACs, rather than GCs, can mediate retinal lamination for the inner retina. For example, despite the null mutants of *atoh7* in mice [27] and zebrafish [28] do not possess GCs, retinal lamination in these mutants is relatively normal. These suggest that a cell type in the INL or the INL itself is essential for establishing retinal lamination. It has been observed that the first ACs extend neuronal projections to form a neuropil, which will ultimately become a laminated IPL, in WT embryos as early as 42 hpf [29] and in the zebrafish mutant that lacks GCs [30]. At the same time, it has been observed that RGC dendrites appear to target pre-laminated amacrine plexuses [31] and that misplaced RGCs always projected dendrites toward nearby neuropil that is formed with amacrine and bipolar cell neurites [32]. These indicate that ACs can potentially give guidance cue for retinal lamination. One possible early-born ACs that may give such guidance cue is the cholinergic ACs [33]. Coincidentally, the anti-Islet1 marker used in this study stains for and Islet1 itself controls the differentiation of cholinergic ACs [34]. Since *irx7* is essential for the neurite outgrowth of these Islet1+ ACs (Figure 5K and L), it is tempting to speculate that *irx7* mediates retinal lamination through the regulation of this process. Nonetheless, ablation of the cholinergic ACs in postnatal ferret by L-glutamate treatment did not seem to affect the formation of other stratifications within the IPL [35]. However, this experimental design did not exclude the possibility that RGC dendrites had already received a cue before the pharmacological ablation of ACs. This can potentially be demonstrated by a specific genetic ablation of cholinergic ACs in the retina.

Much less is known about the regulation of the formation of OPL. While this study did not directly investigate the neuronal projections into the OPL, the observation that Irx7 knockdown also compromised the OPL in a way analogous to the IPL (Figure 5C and D) suggests that the OPL may also have a lower number of neuronal projections. Again, MCs do not seem to be necessary in the establishment and stabilization of the newly formed cone synapses [26], or do they, or BCs, born early enough to mediate IPL formation. There are also evidences that cones are not playing a major role in retinal lamination. For example, the overexpression of NRL, a TF that specifies rod fate, can lead to the production of an all rods retina in mice without altering retinal lamination [36]. Also, it has been shown that even though cone terminals are stratified before HC stratification in mice, the dendritic stratification of HC are not affected in coneless transgenic mice [37]. Nonetheless, cones afferents do play a role in refining the connection with HCs because it has also been noticed that the proper dendritic branching and terminal clustering of HCs within the OPL is dependent on the cone afferents. Together, these observations suggest that HCs may play a role in mediating the formation of OPL. Intriguingly, *ptf1a* is transiently expressed during the development of all HCs and ACs, and quickly turned off after terminal division [38] and *Ptf1a's* function is essential for the determination of HCs and GCs [39], [40], [41], [42]. Since *ptf1a* is over-expressed in the Irx7-deficient retinas at both 52 (Figure 8I and J) and 72 hpf (Figure 8K and L), it is possible that *irx7* represses *ptf1a* and in turn allows for a proper differentiation of both ACs and HCs to mediate retinal lamination. While there are reports that show either suppression [40], [42] or overexpression [39], [41] of *Ptf1a* disrupts retinal lamination, these studies also demonstrate that the *Ptf1a* levels determines ACs and HCs and yields a corresponding change in their numbers. However, ACs and HCs number were not increased in the *ptf1a* overexpression caused by Irx7 knockdown. One possible reason for this difference is that the overexpression of *ptf1a* in these other studies starts at an earlier stage of development (E2 in chick by virus infection [41] and stage 22 in frog by dexamethasone induction of an inducible construct [39]), while in the current study the overexpression is an indirect outcome of Irx7 knockdown. Furthermore, Irx7's expression wave spreads through the zebrafish retina (Figure 1) slightly later than the documented *ptf1a*'s expression [38] which completes very quickly between 35 and 40 hpf. It is possible that the ACs and HCs specification driven by *ptf1a* mostly takes place during this period and the subsequent control of *ptf1a* expression by *irx7* mediates another functional role of *ptf1a*.

It should be noted that despite a specific knockdown of Irx7, the retinal lamination has not been completely eliminated (Figure 5A–D). One obvious possibility is that knockdown is transient and incomplete, and the residual Irx7 can mediate a partial formation of the retinal lamination. Alternatively, there is an independent genetic circuit that can also mediate this process. A likely candidate for this would be Shha.

The expression of Irx7 in the prospective INL is essential for photoreceptors differentiation Despite its exclusive expression in the INL, *irx7* is also essential for the photoreceptors differentiation (Figure 6). The regulation of this differentiation circuit is likely through the transcriptional regulation of TFs that specify photoreceptors, including *crx*, *neurod*, *nr2e3* and *nrl* (Figure 9 and 11) [43]. In this study, the expression of *crx* and *neurod* in the ONL was generally lower at both 52 and 72 hpf in Irx7-deficient retinas (Figure 9). The decrease of their expression and the potential attenuation of their functions are consistent with the observed defects in photoreceptor differentiation (Figure 6). Interestingly, the differentiation problems found in the Irx7 morphants are similar to that in the Crx knockdown, in which photoreceptors are immature, all *opsins* are down-regulated, *vsx1*'s expression is suppressed at 48 hpf but become relatively normal by 72 hpf, and the cell cycle withdrawal is delayed [44]. These suggest that the photoreceptor phenotypes in the morphants are very likely mediated through the transcriptional regulation of *crx.* Both *nrl* and *nr2e3*, TFs that specify rod fate [45], were substantially up-regulated in the ONL of the morphants at 72 hpf (Figure 9). This suggests that Irx7 normally suppresses these TFs and that there is a potential rod fate bias in Irx7-deficient retinas. However, such bias was not observed (Figure 6). This may be due to the suppression of upstream regulators *crx* and *neurod* which compromised the permissive environment for rod overproduction.

Since *irx7* only expresses in the prospective INL, this restricts the location at which the genetic interaction with these photoreceptor TFs can occur. There are at least two non-exclusive possibilities. First, *irx7* can co-express in the precursors that express these TFs. For example, this study and others have demonstrated that there is expression of *crx*, *neurod* and *nr2e3* in INL at 52 hpf (Figure 9) [46], [47], when *irx7* is expressing extensively in the same region. While not characterized in this study, it is possible that these TFs may co-express in the same INL cells. A potential candidate would be the INL progenitors that will ultimately generate rods [48], [49]. Nonetheless, it has been shown that these progenitors can still undergo division, while the *irx7+* cells are post-mitotic (Figure 2), thus this argues against the possibility. Second, retinal progenitors that will begin to withdraw from the cell cycle at 48 hpf and form the ONL [21] may come in close proximity of the *irx7*+ cells in the prospective INL by interkinetic nuclear migration, a process through which the cell body of the progenitors will migrate between the apical and basal surfaces of the retina [50].

### *Irx7*-regulatory network for zebrafish retinal differentiation and implications

*Irx7* was originally identified as a promising candidate in the Smarca4-regulated network that might regulate retinal differentiation and lamination [8], which is defined in the current study. Further, a tentative gene regulatory network through which Irx7 exerts its functions is established (Figure 11). Additional mutual regulatory details on those factors that were analyzed in this study have been incorporated from research on zebrafish and other model systems (Figure S2). This is by no means an exhaustive attempt to build a comprehensive network but rather a beginning step of establishing a global gene network for retinal development. An additional investigation on the regulation of Smarca4-regulated differentiation genes [17] by *irx7* is currently in progress, and will further expand the coverage of the network. It should be noted that the current Irx7-regulatory network has a static topology and has not incorporated and/or addressed many dynamical aspects of gene regulation including expression level, temporal and spatial information. Nonetheless, the knowledge on gene regulatory network for retinal development will lay down the foundation that may aid designing new strategies to treat retinal degeneration and regenerating damaged retinas by guiding therapeutic stem cells to differentiate properly.

## Materials and Methods

### Fish maintenance

Zebrafish, WT AB and *Tg(gfap:GFP)^mi2001^*, were maintained according to standard procedures [51]. All protocols were approved by the Purdue Animal Care and Use Committee.

### Embryo collection

Parental fish were bred for 10 minutes before collection to ensure all embryos would be collected at a similar stage. Embryo staging was done as described [52]. Some embryos were treated with 0.003% PTU (Sigma) in E3 medium [53] between 12 and 23 hpf to prevent melanization. All embryos collected at appropriate stages were fixed and stored as described [17].

### *In situ* hybridization

*In situ* hybridization was performed as described [17]. The riboprobes that were used in this study are as follows: *atonal homolog 7* (*atoh7*); *cone-rod homeobox* (*crx*); *iroquois homeobox protein 7 (irx7), neurogenic differentiation* (*neurod*); *neural retina leucine zipper* (*nrl*); *nuclear receptor subfamily 2 group E member 3* (*nr2e3*); *opsin 1 (cone pigments)*, *short-wave-sensitive 1 (opn1sw1)*; *opsin 1 (cone pigments)*, *short-wave-sensitive 2* (*opn1sw2*); *opsin 1 (cone pigments), long-wave-sensitive 1* (*opn1lw1*), *pancreas specific transcription factor 1a* (*ptf1a*); *patched1 (ptc1); rhodopsin* (*rho*); *visual system homeobox 1 homolog*, *chx10-like* (*vsx1*); *visual system homeobox 2* (*vsx2*). The original references for these probes are listed in File S3.

### Immunohistochemistry

Immunohistochemistry was performed on 10-µm thick cryosections as described [8]. The antibodies used in this study and their dilutions are as follows: mouse anti-zn8 (1∶500, ZIRC), mouse anti-Islet1 (1∶50, Developmental Studies Hybridoma Bank), rabbit anti-PKC βI (PKC) (1∶300, Santa Cruz), mouse anti-Glutamine Synthetase (GS) (1∶500, Millipore), mouse anti-zpr1 (1∶200, ZIRC), mouse anti-zpr3 (1∶200, ZIRC), rabbit anti-phospho-Histone H3 (PH3) (1∶500, Millipore), mouse anti-BrdU (1∶100, Roche), rabbit anti-active caspase3 (1∶500, BD Biosciences), Alexa Fluor 488/555 goat anti-rabbit/mouse IgG (1∶1000, Invitrogen). Alexa Fluor 633 phalloidin (Invitrogen) was included in the first antibody to stain for F-actin. The original references for these antibodies are listed in File S3.

### 5-Bromo-2-deoxyuridine (BrdU) incorporation experiments

Embryos were incubated in 10 mM BrdU (Sigma) and 1% DMSO in E3 medium. To detect the BrdU that were incorporated into the DNA of the cells that had undergone S phase, immunohistochemistry of cryosectioned embryos was performed as described [8], except the cryosections were first incubated with 2N HCl for 1.5 hours and washed with 1× Phosphate buffered saline (PBS) extensively before the blocking step.

### Double labeling by in situ hybridization and immunostaining

*Irx7 in situ* hybridization with anti-Islet1 or anti-GS whole-mount immunostaining was performed as follows: on the first day, embryos were hybridized with the anti-*irx7* probe (DIG-labeled) using the same procedures as in the regular *in situ* hybridization [17], except the embryos used for *irx7* and Islet1 co-staining were not digested with proteinase K. On the second day, the embryos went through the regular stringency washes and then incubated with a mixture of anti-DIG and anti-Islet1 or GS in the blocking solution for overnight at 4°C. On the third day, *irx7* expression was detected by the regular signal detection steps for *in situ* hybridization. Then, embryos were washed extensively in PBST and blocked again with 5% normal goat serum (Sigma) and 1% Triton-X 100 before incubated with the appropriate secondary antibodies against anti-Islet1/GS for overnight at 4°C. Finally, embryos were cryosectioned and imaged by fluorescent microscopy. In addition, embryos used for *irx7 in situ* hybridization and BrdU immunostaining were treated with 10 mM BrdU from 60 to 72 and 72 to 80 hpf. *In situ* hybridization for *irx7* was first conducted regularly as above, and then the embryos were cryosectioned before proceeding to the normal BrdU immunostaining staining procedure as described. The resulting embryos were imaged by fluorescent microscopy.

### Morpholinos (MOs) and mRNA injections

All MOs used in this study for Irx7 (Genbank accession number: BC095012) knockdown were purchased from Gene Tools and are listed in File S3. *Irx7* and *EGFP* mRNA was transcribed from linearized *Irx7-pCS2* [10] with mMessage mMachine Kit (Ambion). All MOs and mRNA were injected into the yolk of embryos at one-cell stage as described [53]. Injection optimization is described in File S3. Three nanograms of *irx7*MO2, which were co-injected with equal amount of *p53*MO (referred to as “*irx7*MO2” in this study), and ten nanograms of *irx7*SMO were chosen for all knockdown experiments. The injection volumes for these two conditions were 1.5 nl and 1.3 nl respectively.

### Anti-Irx7 antibody generation and characterization

Two antigenic peptide sequences: KESDKSDTLTKRESYKQI (corresponding to amino acids 234–251, named Irx7-234) and WPSRDSYSPVNLSTHDLLKQSQ (corresponding to amino acids 293–314, named Irx7-293) were selected to generate a rabbit polyclonal antibody against Irx7 (PRF&L). Additional peptide selection criteria and resulting antibody characterization are shown in File S3 and Figure 4. The cloning and expression conditions of the recombinant proteins that were used in antibody characterization are shown in File S3 and Table S1 respectively.

### Cyclopamine treatment

AB WT embryos were treated with 100 µM cyclopamine (LC laboratories) in 1% ethanol or just 1% ethanol as controls starting at 24, 26, 30 and 36 hpf. All embryos were collected at 52 hpf for *in situ* hybridization analysis.

### Image acquisition and analysis

Bright-field and fluorescent images were acquired by a SPOT-RT3™ colour slider camera (Diagnostic Instruments) mounted on an Olympus BX51 fluorescence compound microscope or SZX16 stereomicroscope. Features of the samples in the images were extracted by i-Solution (IMT i-Solution).

### Statistical analysis and data visualization

All descriptive statistics and data analyses were performed in the R version 2.11.1 (http://www.r-project.org). All raw data and further descriptions on the details of the analyses can be found in File S1 and Table S2. Gene regulatory network was constructed using BioTapestry [54].

## Supporting Information

Figure S1 **Irx7 knockdown compromises differentiation of MCs.** Ten nanograms of control MO and *irx7*SMO was injected into *Tg*(*gfap*:*GFP*)*^mi2001^* embryos. Expression level of the GFP in the retinas was examined in at 59 and 72 hpf. The red arrowheads indicate GFP+ cells, except for the morphant at 59 hpf, which indicate a comparable region of the retina as the control. The retinal area is highlighted by a dotted yellow line. Scale bar = 20 µm.
(TIF)

Figure S2 **A comprehensive Irx7 gene regulatory network for zebrafish retinal development.** A gene regulatory network was constructed using the expression patterns of *irx7* downstream targets as characterized in this study, as well as the mutual interactions of these targets from zebrafish and/or other organisms that are found in the literature (File S2). The specification circuit of the network consists of TFs that specify different retinal cell types while the differentiation circuit consists of genes that carry out cell type specific functions. For example, *opsins* in the photoreceptors are responsible for visual signal transduction. Genes that have not been fully characterized yet are represented by a generic gene (*cell type*-*genes*) in the differentiation circuit. The activation of these “*cell type*-genes” by *irx7*, as well as by other TFs, symbolizes the differentiation of the corresponding cell types driven by the specification circuit. For GCs, an additional *“GC genes for dendritic outgrowth”* is created to distinguish the specific effects of *irx7* knockdown on their dendritic outgrowth (Figure 5). If the actual location of the interaction is not well defined, the domain/cell type in which the effector gene is expressed will be used. The nodes “INL progenitors” and “rod precursors” represent cells that will ultimately migrate to the ONL and give rise to rods [48], [49]. In addition, different retinal regions, including, GCL, INL and ONL can have cellular interaction (≪-≫) that can trigger signal transduction and in turn modulate gene expression. Note that the network topology is a static global view which consists of information obtained from different stages and studies, thus some genes may have both positive and negative inputs if the regulation is dynamical during development. See File S2 for supporting evidence of the connections.
(TIF)

File S1 **Statistical analysis appendix.**
(DOCX)

File S2 **Supporting evidence for the connections in the** ***irx7*** **gene regulatory network.** Experimental evidence was obtained either from this study or from literature. Each edge (regulator-effector) is described by the Regulation type, Connection type (for *irx7* only), Domain in the retina and Stage analyzed (for *irx7* only). In addition, further references for the edge from different animal models are listed. The same regulation type for an edge at different stages is represented by one connector (−> or −|) in Figure 7, while different regulation types for an edge are always represented by a different connector in the same diagram, regardless of the stage. The differentiation circuit in different cell types is represented by a generic gene “*cell type-genes*”.
(DOCX)

File S3 **Supplementary materials and methods.**
(DOCX)

Table S1 **Expression conditions of recombinant Irx7 proteins.** The expression and tested conditions of various recombinant Irx7 proteins are listed. These proteins were used to confirm the specificity of the Irx7 peptide antibodies (File S3).
(DOCX)

Table S2 **Count data for** ***in situ*** **hybridization experiments.** The count distribution of specific staining patterns for different riboprobes in uninjected embryos, controls and morphants at 52 and/or 72 hpf. The most common pattern for the controls and *irx7*SMO morphants are presented in Figure 6, 8 and 9.
(XLSX)
