# Supplementary figures and images for: The Expression of irx7 in the Inner Nuclear Layer of Zebrafish Retina Is Essential for a Proper Retinal Development and Lamination
Source: PLoS One. 2012 Apr 23;7(4):e36145. doi: 10.1371/journal.pone.0036145 (PMC3335143; doi:10.1371/journal.pone.0036145)

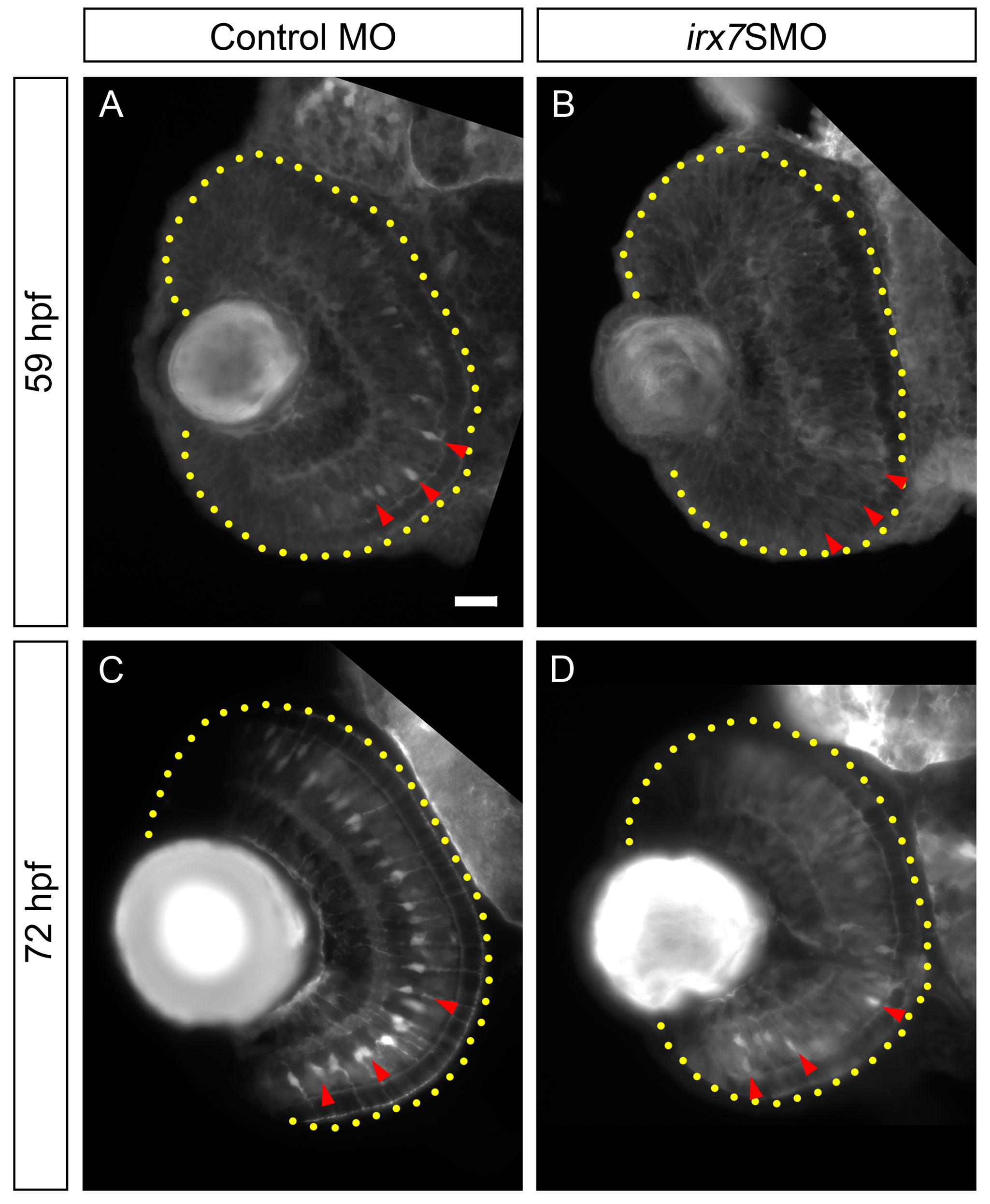

Supplement: Figure S1 — Irx7 knockdown compromises differentiation of MCs. Ten nanograms of control MO and irx7SMO was injected into Tg(gfap:GFP)mi2001 embryos. Expression level of the GFP in the retinas was examined in at 59 and 72 hpf. The red arrowheads indicate GFP+ cells, except for the morphant at 59 hpf, which indicate a comparable region of the retina as the control. The retinal area is highlighted by a dotted yellow line. Scale bar = 20 µm. (TIF) [file pone.0036145.s001.tif]

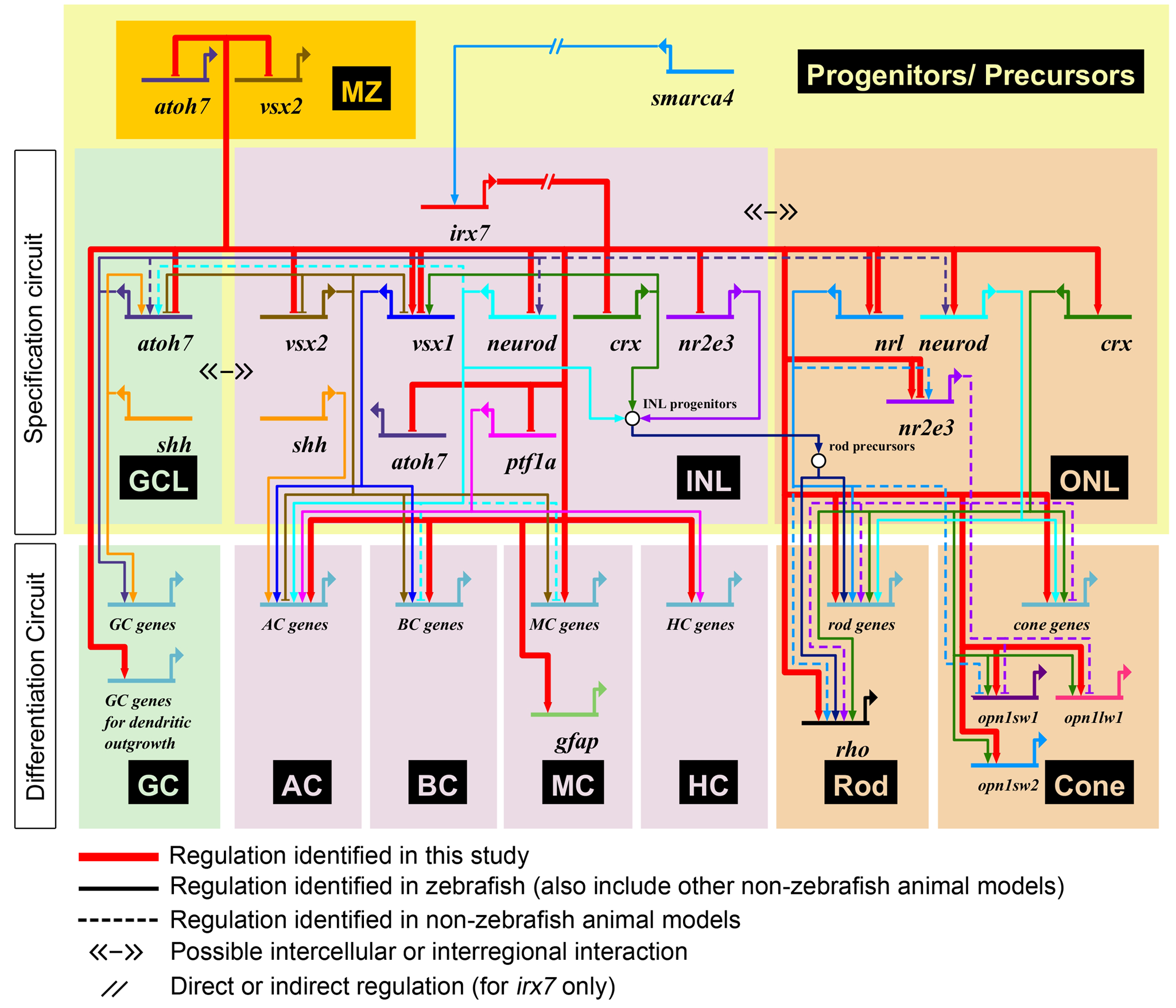

Supplement: Figure S2 — A comprehensive Irx7 gene regulatory network for zebrafish retinal development. A gene regulatory network was constructed using the expression patterns of irx7 downstream targets as characterized in this study, as well as the mutual interactions of these targets from zebrafish and/or other organisms that are found in the literature (File S2). The specification circuit of the network consists of TFs that specify different retinal cell types while the differentiation circuit consists of genes that carry out cell type specific functions. For example, opsins in the photoreceptors are responsible for visual signal transduction. Genes that have not been fully characterized yet are represented by a generic gene (cell type-genes) in the differentiation circuit. The activation of these “cell type-genes” by irx7, as well as by other TFs, symbolizes the differentiation of the corresponding cell types driven by the specification circuit. For GCs, an additional “GC genes for dendritic outgrowth” is created to distinguish the specific effects of irx7 knockdown on their dendritic outgrowth (Figure 5). If the actual location of the interaction is not well defined, the domain/cell type in which the effector gene is expressed will be used. The nodes “INL progenitors” and “rod precursors” represent cells that will ultimately migrate to the ONL and give rise to rods [48], [49]. In addition, different retinal regions, including, GCL, INL and ONL can have cellular interaction (≪-≫) that can trigger signal transduction and in turn modulate gene expression. Note that the network topology is a static global view which consists of information obtained from different stages and studies, thus some genes may have both positive and negative inputs if the regulation is dynamical during development. See File S2 for supporting evidence of the connections. (TIF) [file pone.0036145.s002.tif]
