# Supplementary material for: The Expression of irx7 in the Inner Nuclear Layer of Zebrafish Retina Is Essential for a Proper Retinal Development and Lamination
Source: PLoS One. 2012 Apr 23;7(4):e36145. doi: 10.1371/journal.pone.0036145 (PMC3335143; doi:10.1371/journal.pone.0036145)
Supplement: File S1 — Statistical analysis appendix. (DOCX) [file pone.0036145.s003.docx]

**File S1. Statistical analysis appendix**

***Table of contents***

**I. Introduction**

1. **General**
2. **Count for specific cell number obtained from immunostaining and area analysis**
3. ***In situ* hybridization analysis**

**II. Descriptions of specific analyses**

******* *The following data are presented in the order as they appear in the Results section of the manuscript, regardless of the type of experimental investigations.*

1. **Linear mixed-effects model**
   1. **for delineating the specific eye size reduction in *irx7*SMO and *irx7*MO2 morphants**
   2. **for determining the reduction of INL thickness in *irx7*SMO morphants.**
2. **MCs analysis**
3. **BCs analysis**
4. **ACs analysis**
5. **HCs analysis**
6. **GCs analysis**
7. **Photoreceptor analysis**
8. **Apoptosis analysis**
9. **Cell Cycle Analysis**
10. ***Irx7* mRNA rescue analysis**
11. **Cyclopamine treatment analysis**

**I. Introduction**

**1. General**

In general, continuous data were summarized by mean ($\bar{x}$) and standard deviation (*s*) if the distribution is normal for all experiment groups in an experiment, otherwise the data were summarized by median (*M*) and median absolute deviation (MAD). The analysis of these data was conducted by Student’s *t*-test for two groups with equal variance, Welch’s *t*-test for two groups with unequal variance and ANOVA for three or more groups, if the basic assumptions of the analyses were fulfilled; otherwise non-parametric alternatives, Wilcoxon and Kruskal-Wallis [1] rank sum tests were used for the analysis of data for two and three groups respectively. *Post hoc* comparisons for ANOVA and Kruskal-Wallis test were conducted using Bonferroni-corrected *t-*test and multiple comparison test after Kruskal-Wallis [2] respectively. The latter procedure is implemented in the package *pgirmess* in R. Count data were analyzed by two-tailed Chi-square or Fisher’s exact tests and *p*-values adjusted by Bonferroni correction if necessary; if the outcome is binary and the data structure did not fit in these tests, logistic regression was conducted as described [3]. Linear mixed-effects model was built to delineate the specific effect on retinal area by MO injections, with the confounding factors including sectioning plane and batches of embryos injected on different days modeled as fixed and random effects respectively [4]. Standard error propagation was used to combine measurement errors of different variables. An alpha level of 0.05 was used for all statistical tests.

**2. Count for specific cell number obtained from immunostaining and area analysis**

The count for specific cell number was obtained by the experimenter and a blind observer who did not know the sample label. The correlation of the counts for the markers between the two observers were at least 0.963, and the *p-*values of the correlation test of the counts for all markers were much smaller than < 0.05, suggesting the counting process was not biased. Then the counts for the same sample were averaged and normalized by the corresponding retinal area. After normalization, the data became continuous data. These data and area data were analyzed as described in the General section above.

**3. *In situ* hybridization analysis**

The *in situ* hybridization pattern of each gene was artificially classified into different categories. A similar approach has been previously used to analyze photoreceptor differentiation defects in zebrafish development [8]. For example, *opsin* staining results (Figure 6) were classified into four categories: A) strong, B) intermediate, C) weak and D) faint (note that there was a correlation between the extent of staining and staining intensity in an embryo). Then, the number of embryos of uninjected embryos, controls and morphants in each category was counted. Finally, a two-tailed Fisher’s exact test was done to determine whether the knockdown of Irx7 had altered the distribution of the count of categories. The *p-*values were adjusted by Bonferroni correction for multiple hypothesis testing. To ensure our quantification is free of bias, the count for specific pattern was obtained by the experimenter and a blind observer who did not know the sample label (Table S2). The experimenter counted the samples directly under the stereomicroscope, while the blind observer checked the high resolution images that were taken immediately after the *in situ* hybridization experiments; thus the total count was different between the observers in a few cases. Then, the same statistical tests were conducted to infer significance and the results between these two observers compared. In short, the results from these analyses are highly concordant. In particular, 39 out of 40, or 97.5% of the tests for the comparison of expression patterns between two sample groups yielded the same conclusions between the two observers (i.e. the *p*-values in both tests were < 0.05 or > 0.05). Moreover, all 20 tests (100%) for the comparison between *irx7*SMO morphants and controls yielded the same conclusion (i.e. the *p*-values in both tests were < 0.05 or > 0.05), supporting the quantification of the expression patterns in this study is free of bias. The data from the experimenter are presented in subsequent analyses and interpretations.

**II. Descriptions of specific analyses**

The following data are presented in the order as they appear in the Results section of the manuscript, regardless of the type of experimental investigations.

**1. Linear mixed-effects model**

**a. for delineating the specific eye size reduction in *irx7*SMO and *irx7*MO2 morphants**

The retinal area of the injected embryos was measured from the sections that cut through the optical nerve from ten independent experiments. For each section, attempts were made to cut through the optic nerves in both eyes at the same time to maximize comparability across sections. There were five experimental conditions in these experiments and the total number of samples in each condition is as follows: irx7SMO (N = 70), control MO (N = 69), irx7MO2 (N = 59) and irx7MO2-6bms (N = 63) morphants, and uninjected embryos (N = 49). Out of 310 sections analyzed, 195 contained optic nerves in both eyes while the remaining 115 contained a complete optic nerve in one eye. To delineate the specific effect on retinal area by Irx7 knockdown and to differentiate that from the potential confounding effects caused by a difference in sectioning plane (i.e. with optic nerve in one vs. both eyes), and intrinsic variation of the size of the embryos collected from different experiments, a linear mixed-effects model was fit. The effect on retinal area caused by Irx7 knockdown and the sectioning plane was modeled as fixed effects, while the different batches of embryos collected from different experiments was modeled as a random effect. The analysis shows that the change in the sectioning plane did not affect the retinal area (*F*(1, 291) = 0.0269, *p-*value = 0.8699) or its variance of the measurements, thus this term was dropped from the model. In the final model, the Irx7 knockdown had a specific effect on retinal area (*F*(4, 291) = 151.64, *p-*value < 0.001). In particular, there was a decrease in the retinal area in the *irx7*SMO and *irx7*MO2 morphants compared to their corresponding controls (*irx7*SMO vs. control MO (ratio of area from the fitted model ± standard error): 72.92 ± 5.81%; *irx7*MO2 vs. *irx7*MO2-6bms: 71.54 ± 6.37%; Bonferroni-adjusted *p-*values < 0.0001 in both cases). In the meantime, there was no difference in the retinal areas between the two morphants, between the two controls, and between the *irx7*MO2-6bms controls and the uninjected embryos (Bonferroni-adjusted *p-*values = 1, 1 and 0.14 respectively). However, there was a slight decrease in retinal area in the control MO injected embryos compared with the uninjected embryos (91.93±5.55%; Bonferroni-adjusted *p-*value = 0.00054), indicating that injection of this amount of the control MO might cause a non-specific reduction in retinal area. Nonetheless, the consistent decrease in retinal areas between the two morphants compared with their corresponding controls suggests that this decrease was a specific outcome of the Irx7 knockdown and that the optimized injection amounts would reveal the specific effect of the knockdown on retinal development.

**b. for determining the reduction of INL thickness in irx7SMO morphants.**

To determine the extent of the thinning of the INL, its thickness was measured from sections collected from three independent experiments (total N = 136 for each of the morphant and control group). To facilitate comparison between sections and experimental conditions, the thicknesses of INL immediately dorsal and ventral to the optic nerve was measured. A linear-mixed effect model was fit for each thickness measurement. The results indicate that the INL thickness was reduced in *irx7*SMO morphant (dorsal INL: *F*(1,63) = 53.25; ventral INL: *F*(1,63) = 62.29; p-values < 0.0001 in both cases).

**2. MCs analysis**

*Anti-GS* (Figure 5E & F)

The number of GS+ MCs per retinal area was different between *irx7*SMO morphants (*M* = 0 μm^-2^, MAD = 0 μm^-2^, N = 7) and controls (*M* = 4.57e-04 μm^-2^, MAD = 1.24e-04 μm^-2^, N = 10) (Kruskal-Wallis rank sum test, chi-squared = 19.83, df = 2, *p*-value = 4.93e-05). The *post hoc* analysis indicates that there were fewer GS+ MCs per retinal area in the *irx7*SMO morphants compared with the controls (adjusted *p*-value < 0.05).

*Tg(gfap:GFP)^mi2001^* (Figure S1)

The initial formation of MCs was analyzed by Irx7 knockdown in the retinas in *Tg(gfap:GFP)^mi2001^* [9], in which the appearance of MC-derived fluorescence has been reported at approximately 48 hpf. The knockdown of Irx7 in *Tg(gfap:GFP)^mi2001^* embryos eliminated the fluorescent signal in the INL at 59 hpf compared with the controls (Figure S1A & B and data not shown). While at 72 hpf, the number of GFP+ MCs per retinal area was still different between *irx7*SMO morphants (*M* = 0.00064 μm^-2^, MAD = 4.43e-04 μm^-2^, N = 10) and controls (*M* = 0.0011 μm^-2^, MAD = 1.88e-04 μm^-2^, N = 10) (Figure S1C & D; Kruskal-Wallis rank sum test, chi-squared = 12.6531, df = 2, *p*-value = 0.0018). The *post hoc* analysis indicates that there was a decrease in the number of GFP+ MCs per retinal area in the *irx7*SMO morphants compared with the controls (adjusted *p*-value < 0.05).

**3. BCs analysis**

*Anti-PKC* (Figure 5G & H)

The number of PKC+ BCs per retinal area was different between *irx7*SMO morphants (*M* = 0 μm^-2^, MAD = 0 μm^-2^, N = 7) and controls (*M* = 0.0016 μm^-2^, MAD = 0.00042 μm^-2^, N = 10) (Kruskal-Wallis rank sum test, chi-squared = 19.32, df = 2, *p*-value = 6.38E-05). The *post hoc* analysis indicates that there were fewer PKC+ BCs per retinal area in the *irx7*SMO morphants compared with the controls (adjusted *p*-value < 0.05).

**4. ACs analysis**

*Anti-Islet1* (Figure 5I-L)

Number of ACs per retinal area. The number of ACs per retinal area in the *irx7*SMO morphants ($\bar{x}$ = 0.00056 µm^-2^, *s* = 0.00015 µm^-2^, N = 7) was reduced compared with the controls ($\bar{x}$ *=* 0.00085 µm^-2^, *s* = 0.00023 µm^-2^, N = 6) (two-tailed Student's *t*-test, *t =* 2.6851, df = 11, *p*-value = 0.021).

ACs projections. The percentage of ACs which neuronal projections (*M* = 0 %, MAD = 0 %, N = 7) was reduced compared with the controls (*M* = 43.1 %, MAD = 7.4 %, N = 6). Indeed, only two out of 16 ACs in one morphant showed signs of neuronal projections, all the remaining retinas had no ACs with projections. A logistic regression analysis indicates that there was a difference in the proportion of ACs with projections between *irx7*SMO morphants and controls (chi-squared = 44.0, df = 1, *p*-value = 3.29e-11). Specifically, Irx7 knockdown substantially reduced the proportion of ACs with projections compared with the controls (coefficient = -3.21, SE = 0.74, Z= -4.354, *p*-value = 1.33e-05).

**5. HCs analysis**

*Anti-Islet1* (Figure 5I-L)

There were fewer Islet1+ HCs per retinal area in the *irx7*SMO morphants ($\bar{x}$ *=* 0.00044 µm^-2^, *s* = 0.00022 µm^-2^, N = 7) compared with the controls ($\bar{x}$ *=* 0.00093 µm^-2^, *s* = 8.58e-05 µm^-2^, N = 6) (two-tailed Welch two sample *t*-test, *t =* 5.5106, df = 8.032, *p*-value = 0.00056).

**6. GCs analysis**

*Anti-zn8* (Figure 5M-T)

There was no difference in the number of zn8+ GCs per retinal area in the *irx7*SMO morphants ($\bar{x}$ *=* 0.0071 µm^-2^, *s* = 0.0012 µm^-2^, N = 9) compared with the controls ($\bar{x}$ *=* 0.0068 µm^-2^, *s* = 0.00067 µm^-2^, N = 7) (two-tailed Welch two sample *t*-test, *t =* -0.6317, df = 13.06, *p*-value = 0.54).

**7. Photoreceptor analysis**

*Anti-zpr1* (Figure 6A-B)

The number of zpr1+ cells per retinal area in the *irx7*SMO morphants (*M* = 0.00048 μm^-2^, MAD = 0.00048 μm^-2^, N = 7) was reduced compared with the controls (*M* = 0.0024 μm^-2^, MAD = 0.00014 μm^-2^, N = 6) (two-tailed Wilcoxon rank sum test, W = 42, *p-*value = 0.0034)

*Anti-zpr3* (Figure 6C-D)

The number of zpr3+ cells per retinal area in the *irx7*SMO morphants (*M* = 0.00043 μm^-2^, MAD = 0.00022 μm^-2^, N = 7) was reduced compared with the controls (*M* = 0.0023 μm^-2^, MAD = 0.00015 μm^-2^, N = 7) (two-tailed Wilcoxon rank sum test, W = 49, *p-*value = 0.00058)

*opn1sw1, opn1sw2, opn1lw1 and rho* (Figure 6E-L)

A two-tailed Fisher’s exact test analysis showed that there was a difference between the count distribution of *irx7*SMO morphants compared with the controls for all *opsins* analyzed (*opn1sw1* - *irx7*SMO morphants: 0, 8, 6, 14 vs. controls: 3, 15, 4, 0. Bonferroni-adjusted *p-*value = 6.48e-05; *opn1sw2* - *irx7*SMO morphants: 0, 4, 3, 6 vs. controls: 2, 11, 5, 0. Bonferroni-adjusted *p-*value = 0.013; *opn1lw1* - *irx7*SMO morphants: 0, 2, 1, 10 vs. controls: 4, 14, 5, 0. Bonferroni-adjusted *p-*value = 6.04e-6; *rho*: *irx7*SMO morphants: 0, 4, 8, 5 vs. controls: 0, 11, 5, 0; Bonferroni-adjusted *p-*value = 0.021). Also, the count distribution of the expression pattern types of all *opsins* between the controls and the uninjected embryos was different (Bonferroni-adjusted *p-*value < 0.05 in all cases).

**8. Apoptosis analysis**

*Anti-active caspase3* (Figure 7A-H)

To detect apoptotic cells, immunostaining of anti-active caspase3 [10] was conducted on retinal sections of morphants at 28, 36, 52 and 72 hpf (N = 10, 13, 10 & 13 respectively). The results were compared with that obtained from corresponding controls (N = 11, 12, 11 & 12 respectively) and uninjected embryos (N = 12, 8, 6 & 7 respectively). All retinas at 28, 36 and 52 hpf have fewer than five active capsase3+ cells per retina. In addition, there was no change in the positive cells per retinal area between all three sample types at these three stages (Figure S7 and data not shown). At 72 hpf, there were a few more active caspase3+ cells in the controls than the other sample types, and the number of active caspase3+ cells per retinal area was different between the uninjected embryos (*M* = 4.1e-05 μm^-2^, MAD = 9.28e-06 μm^-2^), controls (*M =* 0.00018 μm^-2^, MAD = 5.36e-05 μm^-2^) and morphants (*M =* 0 μm^-2^, MAD = 0 μm^-2^) (Figure S7D & H; Kruskal-Wallis rank sum test, chi-squared = 17.83, df = 2, *p*-value = 0.00013). The *post hoc* analysis indicates that there was an increase in the number of active caspase3+ cells per retinal area in the controls compared with morphants (adjusted *p-*value < 0.05), but there was no difference between the controls and uninjected embryos, or between the morphants and uninjected embryos (adjusted *p-*value > 0.05 in both cases). While the results show that control MO injection slightly induced apoptosis at 72 hpf, they argue against the role of apoptosis in the eye size reduction of morphants.

**9. Cell Cycle Analysis**

*Anti-PH3* (Figure 7I-K)

The anti-PH3 staining was performed on uninjected embryos, controls and morphants collected at 28, 36, 52 and 72 hpf (For each stage, uninjected embryos: N = 7, 7, 16, 13; controls: N = 9, 7, 13, 16; *irx7*SMO morphants: N = 7, 7, 13, 13). There was no difference in the number of PH3+ cells per retinal area as well as the staining pattern between controls and morphants at 28, 36 and 52 hpf (data not shown). While at 72 hpf, additional PH3+ cells were identified in the apical retina, an ectopic location for them at this stage, in the morphants (Figure 4B, yellow arrowheads) compared with the controls (Figure 4A). The number of PH3+ cells per retinal area was different between the uninjected embryos (*M =* 0.00022 μm^-2^, MAD = 0.00013 μm^-2^), control (*M =* 0.00020 μm^-2^, MAD = 8.05e-05 μm^-2^) and morphants (*M =* 0.0012 μm^-2^, MAD = 0.00042 μm^-2^) (Figure 4C; Kruskal-Wallis rank sum test, chi-squared = 26.3357, df = 2, *p*-value = 1.91e-06). The *post hoc* analysis indicates that there was an increase in the number of PH3+ cells per retinal area in the morphants compared with controls and uninjected embryos (adjusted *p-*value < 0.05 in both comparisons) but not among the latter two groups (adjusted *p-*value > 0.05).

*Anti-BrdU* (Figure 7L-Q)

The BrdU+ area was traced in the image and was normalized by the total retinal area (36-52hpf treatment group: uninjected embryos (*M =* 67.72%, MAD = 2.48%, N = 7), controls (*M =* 68.78%, MAD = 7.94%, N = 7) and morphants (*M =* 93.03%, MAD = 2.89%, N = 7) (Figure 4F); 52-72 hpf treatment group: uninjected embryos (*M =* 18.40%, MAD = 6.66%, N = 11), controls (*M =* 22.42%, MAD = 5.69%, N = 10) and morphants (*M =* 62.58%, MAD = 4.13%, N = 8) (Figure 4I)). There was a difference in the percentage of BrdU+ area in the retinas between different conditions in both BrdU treatment groups (Kruskal-Wallis rank sum test, 36-52 hpf group: chi-squared = 13.78, df = 2, *p*-value = 0.0010; 52-72 hpf group: chi-squared = 18.58, df = 2, *p*-value = 9.23e-05). The *post hoc* analysis indicates that there was an increase in the BrdU+ area in the morphants compared with controls and uninjected embryos (adjusted *p-*value < 0.05 in both comparisons), but not among the latter two groups (adjusted *p-*value > 0.05).

**10. *Irx7* mRNA rescue analysis**

*Anti-zpr1* (Figure 10A & B)

Despite the median of the zpr1+ cells per retinal area in the rescued *irx7*SMO retinas was noticeably larger than (*M* = 0.0013 μm^-2^, MAD = 0.0012 μm^-2^, N = 10) the *irx7*SMO morphants (*M* = 0.00021 μm^-2^, MAD = 0.00031 μm^-2^, N = 9), a two-tailed Wilcoxon rank sum test indicated that there was no difference between the two groups (W = 25, *p-*value = 0.12). The main reason is that the phenotypic variation in the rescued embryos was large. Even though quite a few embryos had more zpr1+ cells in the central retina of the rescued group that was not observed in the morphant group, there were also a few embryos in the rescued group that number of zpr1+ cells was comparable to the morphant group; hence a lack of significance in this statistical test.

*Anti-zpr3* (Figure 10C & D)

The number of zpr3+ cells per retinal area in the rescued *irx7*MO2 morphants (*M* = 0.00083 μm^-2^, MAD = 0.00025 μm^-2^, N = 9) was higher compared with the *irx7*MO2 morphants (*M* = 0.00044 μm^-2^, MAD = 0.00015 μm^-2^, N = 9) (two-tailed Wilcoxon rank sum test, W = 16, *p-*value = 0.031)

**11. Cyclopamine treatment analysis**

The Shha signal transduction was inhibited by treating the embryos with cyclopamine, a Shh inhibitor [11], starting at 24, 26, 30 and 36 hpf. The embryos were collected at 52 hpf and the *irx7* expression pattern in the retinas (regions I-V; as defined in Figure 1H) detected by *in situ* hybridization. The number of embryos with *irx7* expression up to a particular region in the cyclopamine-treated, ethanol (carrier)-treated and untreated embryos was counted. All treatment lengths yielded similar results in different trials, and the longest one, the 24-52 hpf group, is discussed here. This longest treatment would give the most drastic results in theory because all known neurogenic waves mediated by Shha in the retina would have been sufficiently inhibited. The results showed that the count distribution of *irx7* expression patterns in the embryos from these experimental groups was not different (cyclopamine-treated: 2, 1, 1, 2, 2; ethanol-treated: 2, 0, 1, 3, 2; untreated: 1, 1, 1, 0, 5; two-tailed Fisher exact test, Bonferroni-adjusted *p-*values for the comparison between ethanol-treated & untreated embryos, and between ethanol- & cyclopamine-treated embryos are 0.60 and 1 respectively).

**References**

1. Kruskal WH, Wallis WA (1952) Use of Ranks in One-Criterion Variance Analysis. J Am Stat Assoc 47: 583-621.

2. Siegel S, Castellan NJ (1988) Nonparametric statistics for the behavioral sciences. New York: McGraw-Hill. xxiii, 399 p. p.

3. Hensley MR, Leung YF (2010) A convenient dry feed for raising zebrafish larvae. Zebrafish 7: 219-231.

4. Neter J (1996) Applied linear statistical models. Chicago: Irwin. xv, 1408 p. p.

5. Schmitt EA, Dowling JE (1996) Comparison of topographical patterns of ganglion and photoreceptor cell differentiation in the retina of the zebrafish, Danio rerio. J Comp Neurol 371: 222-234.

6. Raymond PA, Barthel LK, Curran GA (1995) Developmental patterning of rod and cone photoreceptors in embryonic zebrafish. J Comp Neurol 359: 537-550.

7. Schmitt EA, Dowling JE (1999) Early retinal development in the zebrafish, Danio rerio: light and electron microscopic analyses. J Comp Neurol 404: 515-536.

8. Bernardos RL, Lentz SI, Wolfe MS, Raymond PA (2005) Notch-Delta signaling is required for spatial patterning and Muller glia differentiation in the zebrafish retina. Dev Biol 278: 381-395.

9. Bernardos RL, Raymond PA (2006) GFAP transgenic zebrafish. Gene Expr Patterns 6: 1007-1013.

10. Maurus D, Harris WA (2009) Zic-associated holoprosencephaly: zebrafish Zic1 controls midline formation and forebrain patterning by regulating Nodal, Hedgehog, and retinoic acid signaling. Genes Dev 23: 1461-1473.

11. Incardona JP, Gaffield W, Kapur RP, Roelink H (1998) The teratogenic Veratrum alkaloid cyclopamine inhibits sonic hedgehog signal transduction. Development 125: 3553-3562.
