# Supplementary material for: The Expression of irx7 in the Inner Nuclear Layer of Zebrafish Retina Is Essential for a Proper Retinal Development and Lamination
Source: PLoS One. 2012 Apr 23;7(4):e36145. doi: 10.1371/journal.pone.0036145 (PMC3335143; doi:10.1371/journal.pone.0036145)
Supplement: File S2 — Supporting evidence for the connections in the irx7 gene regulatory network. Experimental evidence was obtained either from this study or from literature. Each edge (regulator-effector) is described by the Regulation type, Connection type (for irx7 only), Domain in the retina and Stage analyzed (for irx7 only). In addition, further references for the edge from different animal models are listed. The same regulation type for an edge at different stages is represented by one connector (−> or −|) in Figure 7, while different regulation types for an edge are always represented by a different connector in the same diagram, regardless of the stage. The differentiation circuit in different cell types is represented by a generic gene “cell type-genes”. (DOCX) [file pone.0036145.s004.docx]

**File S2. Supporting evidence for the connections in the *irx7* gene regulatory network.**

| **Edge (regulator-effector)** | **Regulation type** | **Connection type*** | **Domain†** | **Stage analyzed (hpf)*** | **Animal model** | **References** |
| --- | --- | --- | --- | --- | --- | --- |
| *irx7-AC genes* | positive | indirect | AC | 72 | zebrafish | This study |
| *irx7-atoh7* | negative | indirect | GCL | 52 | zebrafish | This study |
| *irx7-atoh7* | negative | indirect | INL | 52 | zebrafish | This study |
| *irx7-atoh7* | negative | indirect | MZ | 52 & 72 | zebrafish | This study |
| *irx7-BC genes* | positive | indirect | BC | 72 | zebrafish | This study |
| *irx7-cone genes* | positive | indirect | Cone | 72 | zebrafish | This study |
| *irx7-crx* | negative | indirect | INL | 52 & 72 | zebrafish | This study |
| *irx7-crx* | positive | indirect | ONL | 52 & 72 | zebrafish | This study |
| *irx7-GC genes for dendritic outgrowth* | positive | Indirect | GC | 72 | zebrafish | This study |
| *irx7-gfap* | positive | indirect | MC | 59 & 72 | zebrafish | This study |
| *irx7-HC genes* | positive | indirect | HC | 72 | zebrafish | This study |
| *irx7-MC genes* | positive | indirect | MC | 72 | zebrafish | This study |
| *irx7-neurod* | negative | indirect | INL | 52 & 72 | zebrafish | This study |
| *irx7-neurod* | positive | indirect | ONL | 52 & 72 | zebrafish | This study |
| *irx7-nr2e3* | negative | indirect | INL | 52 | zebrafish | This study |
| *irx7-nr2e3* | positive | indirect | ONL | 52 | zebrafish | This study |
| *irx7-nr2e3* | negative | indirect | ONL | 72 | zebrafish | This study |
| *irx7-nrl* | positive | indirect | ONL | 52 | zebrafish | This study |
| *irx7-nrl* | negative | indirect | ONL | 72 | zebrafish | This study |
| *irx7-opn1sw1* | positive | indirect | ONL | 72 | zebrafish | This study |
| *irx7-opn1sw2* | positive | indirect | ONL | 72 | zebrafish | This study |
| *irx7-opn1lw1* | positive | indirect | ONL | 72 | zebrafish | This study |
| *irx7-ptf1a* | negative | indirect | INL | 52 & 72 | zebrafish | This study |
| *irx7-rho* | positive | indirect | ONL | 72 | zebrafish | This study |
| *irx7-rod genes* | positive | indirect | Rod | 72 | zebrafish | This study |
| *irx7-vsx1* | positive | indirect | INL | 52 | zebrafish | This study |
| *irx7-vsx1* | negative | indirect | INL | 72 | zebrafish | This study |
| *irx7-vsx2* | negative | indirect | MZ | 52 & 72 | zebrafish | This study |
| *irx7-vsx2* | negative | indirect | INL | 72 | zebrafish | This study |
| *atoh7-atoh7* | positive | - | GCL | - | medaka  chick  chick  chick | [1]  [2]  [3]  [4] |
| *atoh7-GC genes* | positive | - | GC | - | zebrafish | [5] |
| *atoh7-neurod* | positive | - | INL/ONL | - | mouse | [6] |
| *crx-rod & cone genes* | positive | - | rod & cone | - | zebrafish  human  rat  mouse | [7]  [8]  [9]  [10] |
| *crx-INL progenitors^‡^* | positive | - | INL | - | zebrafish | [11] |
| *crx-rho* | positive | - | rod | - | zebrafish | [7] |
| *crx-opn1lw1* | positive | - | cone | - | zebrafish | [7] |
| *crx-opn1sw1* | positive | - | cone | - | zebrafish | [7] |
| *crx-opn1sw2* | positive | - | cone | - | zebrafish | [7] |
| *INL progenitors – rod precursors^‡^* | migration | - | INL – ONL | - | zebrafish | [11,12,13] |
| *neurod-AC genes* | positive | - | AC | - | zebrafish  rat | [14]  [15] |
| *neurod-atoh7* | positive |  | GC | - | chick | [2] |
| *neurod-BC genes* | negative | - | BC | - | rat | [15] |
| *neurod-cone genes* | positive | - | cone | - | zebrafish  mouse | [14]  [16] |
| *neurod-MC genes* | negative | - | MC | - | rat | [15] |
| *neurod- INL progenitors^‡^* | positive | - | INL | - | zebrafish | [11] |
| *neurod-rod genes* | positive | - | Rod | - | zebrafish  zebrafish  mouse  rat | [14]  [11]  [16]  [15] |
| *nr2e3-cone genes* | negative | - | Cone | - | mouse | [17,18] |
| *nr2e3-opn1sw1* | negative | - | Cone | - | mouse | [17,18] |
| *nr2e3-opn1lw1* | negative | - | Cone | - | mouse | [17,18] |
| *nr2e3-rod genes* | positive | - | Rod | - | mouse | [17,18] |
| *nr2e3-rho* | positive | - | Rod | - | mouse | [17,18] |
| *nr2e3-INL progenitors^‡^* | positive | - | INL | - | zebrafish | [11] |
| *nrl-rho* | positive | - | Rod | - | mouse | [19] |
| *nrl-nr2e3* | positive | - | INL | - | mouse | [19,20] |
| *nrl-opn1sw1* | negative | - | Cone | - | mouse | [19] |
| *nrl-rod genes* | positive | - | Rod | - | zebrafish  mouse | [11]  [19] |
| *ptf1a-AC genes* | positive | - | AC | - | zebrafish | [21] |
| *ptf1a-HC genes* | positive | - | HC | - | zebrafish | [21] |
| *smarca4-irx7* | positive | - | INL | 36 & 52 | zebrafish | [22,23] |
| *rod precursors^‡^ - rod genes* | positive | - | Rod | - | zebrafish | [11,12,13] |
| *rod precursors^‡^ - rho* | positive | - | Rod | - | zebrafish | [11] |
| *shh-AC genes* | positive | - | AC | - | zebrafish | [24] |
| *shh-atoh7* | positive | - | GCL | - | zebrafish | [25]  [26] |
| *shh-GC genes* | positive | - | GC | - | zebrafish | [27] |
| *vsx1-AC genes* | positive | - |  | - | zebrafish | [28] |
| *vsx1-BC genes* | positive | - |  | - | zebrafish  mouse | [28]  [29] |
| *vsx2-atoh7* | negative | - | GCL | - | zebrafish | [28] |
| *vsx2-AC genes* | negative | - | AC | - | zebrafish | [28] |
| *vsx2-BC genes* | positive | - | BC | - | zebrafish | [28] |
| *vsx2-MC genes* | positive | - | MC | - | zebrafish | [28] |
| *vsx2-vsx1* | negative | - | INL | - | zebrafish | [28] |
| *vsx2-vsx2* | negative | - | INL | - | zebrafish | [28] |

^*^ for *irx7* edges only; the regulation on the “*cell-type* genes” in the differentiation circuit are inferred by the immunostaining observations, thus the associated stage is at 72 hpf.

**^†^** corresponds to the domains used in Figure 7. If the actual interaction location is not well defined, the domain in which the effector gene is expressed and/or the cell type in which the effector gene mediates its developmental roles will be used.

^‡^ Both INL progenitors and rod precursors are cell states defined by [11,12,13]. INL progenitors migrate to the ONL and form rod precursors which ultimately become rod photoreceptors.

**References**

1. Del Bene F, Ettwiller L, Skowronska-Krawczyk D, Baier H, Matter JM, et al. (2007) In vivo validation of a computationally predicted conserved Ath5 target gene set. PLoS Genet 3: 1661-1671.

2. Matter-Sadzinski L, Matter JM, Ong MT, Hernandez J, Ballivet M (2001) Specification of neurotransmitter receptor identity in developing retina: the chick ATH5 promoter integrates the positive and negative effects of several bHLH proteins. Development 128: 217-231.

3. Skowronska-Krawczyk D, Ballivet M, Dynlacht BD, Matter JM (2004) Highly specific interactions between bHLH transcription factors and chromatin during retina development. Development 131: 4447-4454.

4. Matter-Sadzinski L, Puzianowska-Kuznicka M, Hernandez J, Ballivet M, Matter JM (2005) A bHLH transcriptional network regulating the specification of retinal ganglion cells. Development 132: 3907-3921.

5. Kay JN, Finger-Baier KC, Roeser T, Staub W, Baier H (2001) Retinal ganglion cell genesis requires lakritz, a Zebrafish atonal Homolog. Neuron 30: 725-736.

6. Ma W, Yan RT, Xie W, Wang SZ (2004) A role of ath5 in inducing neuroD and the photoreceptor pathway. The Journal of neuroscience : the official journal of the Society for Neuroscience 24: 7150-7158.

7. Shen YC, Raymond PA (2004) Zebrafish cone-rod (crx) homeobox gene promotes retinogenesis. Dev Biol 269: 237-251.

8. Freund CL, Gregory-Evans CY, Furukawa T, Papaioannou M, Looser J, et al. (1997) Cone-rod dystrophy due to mutations in a novel photoreceptor-specific homeobox gene (CRX) essential for maintenance of the photoreceptor. Cell 91: 543-553.

9. Furukawa T, Morrow EM, Cepko CL (1997) Crx, a novel otx-like homeobox gene, shows photoreceptor-specific expression and regulates photoreceptor differentiation. Cell 91: 531-541.

10. Furukawa T, Morrow EM, Li T, Davis FC, Cepko CL (1999) Retinopathy and attenuated circadian entrainment in Crx-deficient mice. Nat Genet 23: 466-470.

11. Nelson SM, Frey RA, Wardwell SL, Stenkamp DL (2008) The developmental sequence of gene expression within the rod photoreceptor lineage in embryonic zebrafish. Dev Dyn 237: 2903-2917.

12. Otteson DC, D'Costa AR, Hitchcock PF (2001) Putative stem cells and the lineage of rod photoreceptors in the mature retina of the goldfish. Developmental biology 232: 62-76.

13. Otteson DC, Hitchcock PF (2003) Stem cells in the teleost retina: persistent neurogenesis and injury-induced regeneration. Vision research 43: 927-936.

14. Ochocinska MJ, Hitchcock PF (2007) Dynamic expression of the basic helix-loop-helix transcription factor neuroD in the rod and cone photoreceptor lineages in the retina of the embryonic and larval zebrafish. J Comp Neurol 501: 1-12.

15. Morrow EM, Furukawa T, Lee JE, Cepko CL (1999) NeuroD regulates multiple functions in the developing neural retina in rodent. Development 126: 23-36.

16. Pennesi ME, Cho JH, Yang Z, Wu SH, Zhang J, et al. (2003) BETA2/NeuroD1 null mice: a new model for transcription factor-dependent photoreceptor degeneration. J Neurosci 23: 453-461.

17. Cheng H, Aleman TS, Cideciyan AV, Khanna R, Jacobson SG, et al. (2006) In vivo function of the orphan nuclear receptor NR2E3 in establishing photoreceptor identity during mammalian retinal development. Human molecular genetics 15: 2588-2602.

18. Peng GH, Ahmad O, Ahmad F, Liu J, Chen S (2005) The photoreceptor-specific nuclear receptor Nr2e3 interacts with Crx and exerts opposing effects on the transcription of rod versus cone genes. Human molecular genetics 14: 747-764.

19. Mears AJ, Kondo M, Swain PK, Takada Y, Bush RA, et al. (2001) Nrl is required for rod photoreceptor development. Nat Genet 29: 447-452.

20. Oh EC, Cheng H, Hao H, Jia L, Khan NW, et al. (2008) Rod differentiation factor NRL activates the expression of nuclear receptor NR2E3 to suppress the development of cone photoreceptors. Brain research 1236: 16-29.

21. Jusuf PR, Harris WA (2009) Ptf1a is expressed transiently in all types of amacrine cells in the embryonic zebrafish retina. Neural Dev 4: 34.

22. Leung YF, Ma P, Link BA, Dowling JE (2008) Factorial microarray analysis of zebrafish retinal development. Proc Natl Acad Sci U S A 105: 12909-12914.

23. Hensley MR, Emran F, Bonilla S, Zhang L, Zhong W, et al. (2011) Cellular Expression of Smarca4 (Brg1)-regulated Genes in Zebrafish Retinas. BMC Dev Biol 11: 45.

24. Shkumatava A, Fischer S, Muller F, Strahle U, Neumann CJ (2004) Sonic hedgehog, secreted by amacrine cells, acts as a short-range signal to direct differentiation and lamination in the zebrafish retina. Development 131: 3849-3858.

25. Stenkamp DL, Frey RA (2003) Extraretinal and retinal hedgehog signaling sequentially regulate retinal differentiation in zebrafish. Dev Biol 258: 349-363.

26. Kay JN, Link BA, Baier H (2005) Staggered cell-intrinsic timing of ath5 expression underlies the wave of ganglion cell neurogenesis in the zebrafish retina. Development 132: 2573-2585.

27. Neumann CJ, Nuesslein-Volhard C (2000) Patterning of the zebrafish retina by a wave of sonic hedgehog activity. Science 289: 2137-2139.

28. Vitorino M, Jusuf PR, Maurus D, Kimura Y, Higashijima S, et al. (2009) Vsx2 in the zebrafish retina: restricted lineages through derepression. Neural Dev 4: 14.

29. Chow RL, Volgyi B, Szilard RK, Ng D, McKerlie C, et al. (2004) Control of late off-center cone bipolar cell differentiation and visual signaling by the homeobox gene Vsx1. Proc Natl Acad Sci U S A 101: 1754-1759.
