# Supplementary material for: The Expression of irx7 in the Inner Nuclear Layer of Zebrafish Retina Is Essential for a Proper Retinal Development and Lamination
Source: PLoS One. 2012 Apr 23;7(4):e36145. doi: 10.1371/journal.pone.0036145 (PMC3335143; doi:10.1371/journal.pone.0036145)
Supplement: File S3 — Supplementary materials and methods. (DOCX) [file pone.0036145.s005.docx]

**File S3. Supplementary materials and methods**

***Table of contents***

**Morpholinos (MOs) and injections optimization**

**mRNA synthesis and injection optimization**

**Cloning**

**Anti-Irx7 antibody generation and characterization**

**Literature references for markers used in this study**

**Morpholinos (MOs) injections optimization**

Two non-overlapping translation blocking MOs: *irx7*MO1 (5’-GCAAACCCCGTTGATGAAGCAGGCA-3’) [1] and *irx7*MO2 (5’-GGCATCCTTACTCCCTGAGCTCTGG-3’) [2], and one splice blocking MO: *irx7*SMO (5’- GTCAAAATACTACTTACAATGTGTG-3’) [3] were used in this study to specifically knock down Irx7 (Genbank accession number: BC095012). A 5-base mismatch MO: *irx7*MO1-5bms (5’-GCAAACCGCCTTCATCAACCAGGCA-3’), and a 6-base mismatch MO: *irx7*MO2-6bms (5’-GGGATCGTTAGTCCGTGACCTCAGG-3’), were used as controls for *irx7*MO1 and *irx7*MO2 respectively; while a standard control MO (5’-CCTCTTACCTCAGTTACAATTTATA-3’) (control MO) was used as the control for *irx7*SMO.

To optimize the concentration of MOs for knock down, 2, 4, 8 and 10 nanograms (ngs) of *irx7*MO1; 1, 2, 3 and 4 ngs of *irx7*MO2; 4, 6, 8 and 10 ngs of *irx7*SMO were tested. Equal amount of *p53*MO (Gene Tools) was co-injected in some experiments to alleviate potential off-target effects of MO injection [4].

**mRNA synthesis and injection optimization**

*Irx7-pCS2* (former name: iro7-pCS2, containing the *irx7* full-length cDNA) [1] was linearized with Not I (NEB) and transcribed with SP6 RNA polymerase using mMessage mMachine Kit (Ambion). A series of concentrations including 1, 2.5, 5, 10, 20, 50 and 80 picograms (pgs) of this mRNA were injected at one-cell stage embryos. It was found that five pgs of the *irx7* full-length mRNA would lead to embryonic death when injected alone while the embryos developed relatively well when co-injected with this amount of mRNA and *irx7*SMO. This suggests that the mRNA was effective because it could specifically compensate the endogenous Irx7 function that was knocked-down by the *irx7*SMO; and this also further supports that the phenotypes as seen in the Irx7 morphants were specific. The final amount used for the analysis was five pgs for rescue experiments. For overexpression experiments, 3-4 pgs of *irx7* mRNA was injected into the yolk at one cell stage, while equal amount of *EGFP* mRNA was injected as control.

**Cloning**

Irx7NL-pET28a, Irx7C-pET21b+, Irx7F-pET21b+ and Irx7F-pET28a constructs were made to express fragments of Irx7 protein (NL: N-terminal, Long; C: C-terminal, F: Full length) in bacteria (Tuner DE3 or Rosetta DE3). To generate Irx7NL-pET28a, *irx7* coding sequence 1-774 bps was amplified using *irx7-pCS2* [2] by PCR and cloned into the pET28a protein expression vector (Novagen) between BamHI and HindIII restriction sites. To generate Irx7C-pET21b+, *irx7* coding sequence 804-942 bps was amplified using *irx7-pME18S-FL3* (Thermos Fisher Scientific) by PCR and cloned into pET21b+ (Novagen) between BamHI and HindIII restriction sites. To generate Irx7F-pET21b+, the full-length Irx7 coding sequence (1-942 bps) was amplified by PCR from *irx7-pME18S-FL3* and cloned into pET21b+. To generate irx7F-pET28a, the *irx7* full-length sequence was released from Irx7F-pET21b+ by NdeI and HindIII restriction, and cloned into pET28a between the NdeI and HindIII restriction sites.

**Anti-Irx7 antibody generation and characterization**

Two antigenic peptide sequences: KESDKSDTLTKRESYKQI (corresponding to amino acids 234-251, named Irx7-234) and WPSRDSYSPVNLSTHDLLKQSQ (corresponding to amino acids 293-314, named Irx7-293) were selected to generate a rabbit polyclonal antibody against Irx7 (Pocono Rabbit Farm and Laboratory, Inc). These two sequences do not overlap with three known domains in Irx7, including the homeodomain (amino acids 103-166), the acidic amino acid rich region (amino acids 177-215) and the Iro-box (amino acids 256-267). Peptide synthesis, antibody generation and purification were conducted by Pocono Rabbit Farm and Laboratory, Inc. Specifically, the two peptides were co-injected into the rabbit and the resulting anti-Irx7 serum was purified separately by Irx7-234 and Irx7-293 peptides to get anti-Irx7-234 and anti-Irx7-293 antibodies respectively.

The resulting antibodies were characterized by Western blot with both embryos lysate [5] collected at 52 and 72 hpf, and Irx7 protein fragments expressed in bacteria using the constructs prepared in the Cloning section (See Table S1 for expression conditions). Specially, the embryo lysate consisted of 30 or 35 dissected embryonic heads. These embryonic lysates and bacteria expressing Irx7 N-terminal long (NL) (recognized by anti-Irx7-234 only), C-terminal (recognized by anti-Irx7-293 only), and Full-length fragments (recognized by both anti-Irx7-234 and anti-Irx7-29) were lysed with 2X Laemmli buffer (2% SDS, 10% glycerol, 5% 2-mercaptoethanol, 0.002% bromophenol blue, 0.125M Tris-HCl, pH 6.8) at 95 °C for 15 minutes. The proteins in the buffer were resolved by SDS-PAGE and transferred to Immuno-Blot PVDF Membrane (BioRad). Then the membrane was washed with 1X PBS, blocked in 0.1% normal goat serum (Sigma) for 1 hour at room temperature, and incubated with rabbit anti-Irx7 (1:100), mouse anti-γ-Tubulin (Sigma, 1:10000), mouse anti-His-tag (Sigma, 1:3000) or mouse anti-T7 tag antibody (Novagen, 1:10000) at 4°C for overnight. The membrane was then washed with 1X PBS five times before incubated with Alexa Fluor® 680 goat anti-mouse or anti-rabbit IgG (Invitrogen) for 1.5 hours at room temperature. Finally, the membrane was washed with 1X PBS five times and imaged with Odyssey 3.0 (LI-COR Biosciences). When tested on embryo lysates, both anti-Irx7-234 and anti-Irx7-293 could detect the Irx7 protein. Since anti-Irx7-234 was relatively specific and the resulting signal was also stronger, it was used in the subsequent analyses (Figure 4).

**Literature references for markers used in this study**

* except *irx7*

I. In situ *hybridization*

| **Riboprobe** | **Cell type(s) recognized** | **References** |
| --- | --- | --- |
| *atoh7* | GCs | [6] |
| *crx* | Rods, cones | [7,8] |
| *neurod* | ACs, rods, cones | [7,8,9] |
| *nrl* | Rods | [10] |
| *nr2e3* | Rods | [11,12] |
| *opn1sw1* | UV cones | [8] |
| *opn1sw2* | Blue cones | [8] |
| *opn1lw1* | Red cones | [8] |
| *ptf1a* | ACs, HCs | [13] |
| *rho* | Rods | [8] |
| *vsx1* | sub-population of BCs, ACs | [14] |
| *vsx2* | sub-population of BCs, MCs | [14] |

II. *Immunohistochemistry*

| **Antibody** | **Cell type(s) recognized** | **References** |
| --- | --- | --- |
| Anti-zn8 | GCs | [15] |
| Anti-Islet1 | GCs, ACs, BCs, HCs | [16] |
| Anti-PKC | BCs | [6] |
| Anti-GS | MCs | [17] |
| Anti-zpr1 | Red-green double cones | [18] |
| Anti-zpr3 | Rods | [18] |
| Anti-PH3 | Mitotic cells | [19] |
| Anti-BrdU | Cells that have gone through S-phase and labeled by BrdU | [19] |
| Anti-active caspase3 | Apoptotic cells | [20] |

**References**

1. Itoh M, Kudoh T, Dedekian M, Kim CH, Chitnis AB (2002) A role for iro1 and iro7 in the establishment of an anteroposterior compartment of the ectoderm adjacent to the midbrain-hindbrain boundary. Development 129: 2317-2327.

2. Lecaudey V, Anselme I, Rosa F, Schneider-Maunoury S (2004) The zebrafish Iroquois gene iro7 positions the r4/r5 boundary and controls neurogenesis in the rostral hindbrain. Development 131: 3121-3131.

3. Stedman A, Lecaudey V, Havis E, Anselme I, Wassef M, et al. (2009) A functional interaction between Irx and Meis patterns the anterior hindbrain and activates krox20 expression in rhombomere 3. Dev Biol 327: 566-577.

4. Robu ME, Larson JD, Nasevicius A, Beiraghi S, Brenner C, et al. (2007) p53 activation by knockdown technologies. PLoS Genet 3: e78.

5. Westerfield M (2000) The zebrafish book : a guide for the laboratory use of zebrafish (Danio rerio). Eugene, OR: University of Oregon Press.

6. Kay JN, Finger-Baier KC, Roeser T, Staub W, Baier H (2001) Retinal ganglion cell genesis requires lakritz, a Zebrafish atonal Homolog. Neuron 30: 725-736.

7. Ochocinska MJ, Hitchcock PF (2007) Dynamic expression of the basic helix-loop-helix transcription factor neuroD in the rod and cone photoreceptor lineages in the retina of the embryonic and larval zebrafish. J Comp Neurol 501: 1-12.

8. Shen YC, Raymond PA (2004) Zebrafish cone-rod (crx) homeobox gene promotes retinogenesis. Dev Biol 269: 237-251.

9. Pennesi ME, Cho JH, Yang Z, Wu SH, Zhang J, et al. (2003) BETA2/NeuroD1 null mice: a new model for transcription factor-dependent photoreceptor degeneration. J Neurosci 23: 453-461.

10. Nelson SM, Frey RA, Wardwell SL, Stenkamp DL (2008) The developmental sequence of gene expression within the rod photoreceptor lineage in embryonic zebrafish. Dev Dyn 237: 2903-2917.

11. Kitambi SS, Hauptmann G (2007) The zebrafish orphan nuclear receptor genes nr2e1 and nr2e3 are expressed in developing eye and forebrain. Gene Expr Patterns 7: 521-528.

12. Chen J, Rattner A, Nathans J (2005) The rod photoreceptor-specific nuclear receptor Nr2e3 represses transcription of multiple cone-specific genes. J Neurosci 25: 118-129.

13. Jusuf PR, Harris WA (2009) Ptf1a is expressed transiently in all types of amacrine cells in the embryonic zebrafish retina. Neural Dev 4: 34.

14. Vitorino M, Jusuf PR, Maurus D, Kimura Y, Higashijima S, et al. (2009) Vsx2 in the zebrafish retina: restricted lineages through derepression. Neural Dev 4: 14.

15. Leung YF, Ma P, Link BA, Dowling JE (2008) Factorial microarray analysis of zebrafish retinal development. Proc Natl Acad Sci U S A 105: 12909-12914.

16. Link BA, Fadool JM, Malicki J, Dowling JE (2000) The zebrafish young mutation acts non-cell-autonomously to uncouple differentiation from specification for all retinal cells. Development 127: 2177-2188.

17. Peterson RE, Fadool JM, McClintock J, Linser PJ (2001) Muller cell differentiation in the zebrafish neural retina: evidence of distinct early and late stages in cell maturation. The Journal of comparative neurology 429: 530-540.

18. Lee J, Willer JR, Willer GB, Smith K, Gregg RG, et al. (2008) Zebrafish blowout provides genetic evidence for Patched1-mediated negative regulation of Hedgehog signaling within the proximal optic vesicle of the vertebrate eye. Dev Biol 319: 10-22.

19. Yamaguchi M, Tonou-Fujimori N, Komori A, Maeda R, Nojima Y, et al. (2005) Histone deacetylase 1 regulates retinal neurogenesis in zebrafish by suppressing Wnt and Notch signaling pathways. Development 132: 3027-3043.

20. Maurus D, Harris WA (2009) Zic-associated holoprosencephaly: zebrafish Zic1 controls midline formation and forebrain patterning by regulating Nodal, Hedgehog, and retinoic acid signaling. Genes Dev 23: 1461-1473.
