# Supplementary material for: The Expression of irx7 in the Inner Nuclear Layer of Zebrafish Retina Is Essential for a Proper Retinal Development and Lamination
Source: PLoS One. 2012 Apr 23;7(4):e36145. doi: 10.1371/journal.pone.0036145 (PMC3335143; doi:10.1371/journal.pone.0036145)
Supplement: Table S1 — Expression conditions of recombinant Irx7 proteins. The expression and tested conditions of various recombinant Irx7 proteins are listed. These proteins were used to confirm the specificity of the Irx7 peptide antibodies (File S3). (DOCX) [file pone.0036145.s006.docx]

| Irx7 peptide | Expression vector | Bacteria | IPTG | Induction time | Culture medium | Peptidase inhibitor | Anti-irx7 antibody Detection | Tag antibody Detection |
| --- | --- | --- | --- | --- | --- | --- | --- | --- |
| Irx7 NL | pET28a | Rosetta DE3 | 1mM | 4 hours | 2×YT  Chloramphenicol (30mg/ml)  Kanamycin (50mg/ml) | cOmplete  Protease  Inhibitor (Roche) | Detected by anti-Irx7-234 | Detected by Anti-His antibody |
| Irx7F | pET28a | Rosetta DE3 | 1mM | 4 hours | 2×YT  Chloramphenicol (30mg/ml)  Kanamycin (50mg/ml) | cOmplete  Protease  Inhibitor (Roche) | Detected by anti-Irx7-234 | Detected by Anti-His antibody |
| Irx7C | pET21b+ | Rosetta DE3 | 1mM | 3 hours | 2×YT  Chloramphenicol (30mg/ml)  Ampicillin (100mg/ml) | None | Detected by anti-Irx7-293 | Detected by anti-T7 antibody |

Table S1. Expression conditions of recombinant Irx7 proteins.
